# Definition of transcriptome-based indices for quantitative characterization of chemically disturbed stem cell development: introduction of the STOP-Tox_ukn_ and STOP-Tox_ukk_ tests

**DOI:** 10.1007/s00204-016-1741-8

**Published:** 2016-05-17

**Authors:** Vaibhav Shinde, Lisa Hoelting, Sureshkumar Perumal Srinivasan, Johannes Meisig, Kesavan Meganathan, Smita Jagtap, Marianna Grinberg, Julia Liebing, Nils Bluethgen, Jörg Rahnenführer, Eugen Rempel, Regina Stoeber, Stefan Schildknecht, Sunniva Förster, Patricio Godoy, Christoph van Thriel, John Antonydas Gaspar, Jürgen Hescheler, Tanja Waldmann, Jan G. Hengstler, Marcel Leist, Agapios Sachinidis

**Affiliations:** 1 Institute of Neurophysiology and Centre for Molecular Medicine Cologne (CMMC), University of Cologne (UKK), Robert-Koch-Str. 39, 50931 Cologne, Germany; 2 Doerenkamp-Zbinden Chair for In Vitro Toxicology and Biomedicine, University of Konstanz, Box: M657, 78457 Constance, Germany; 3 Konstanz Graduate School Chemical Biology KORS-CB, University of Konstanz, 78457 Constance, Germany; 4 Department of Statistics, TU Dortmund University, Dortmund, Germany; 5 Leibniz Research Centre for Working Environment and Human Factors at the Technical, University of Dortmund (IfADo), Ardeystrasse 67, 44139 Dortmund, Germany; 6 Institute of Pathology, Charité Universitätsmedizin, 10117 Berlin, Germany; 7 Integrative Research Institute for the Life Sciences, Institute for Theoretical Biology, Humboldt Universität, 10115 Berlin, Germany; 8 Centre for Organismal Studies, Heidelberg University, 69120 Heidelberg, Germany

**Keywords:** Human stem cells, Transcriptome, Genomics biomarkers, Developmental toxicity, In vitro test systems

## Abstract

Stem cell-based in vitro test systems can recapitulate specific phases of human development. In the UKK test system, human pluripotent stem cells (hPSCs) randomly differentiate into cells of the three germ layers and their derivatives. In the UKN1 test system, hPSCs differentiate into early neural precursor cells. During the normal differentiation period (14 days) of the UKK system, 570 genes [849 probe sets (PSs)] were regulated >fivefold; in the UKN1 system (6 days), 879 genes (1238 PSs) were regulated. We refer to these genes as ‘developmental genes’. In the present study, we used genome-wide expression data of 12 test substances in the UKK and UKN1 test systems to understand the basic principles of how chemicals interfere with the spontaneous transcriptional development in both test systems. The set of test compounds included six histone deacetylase inhibitors (HDACis), six mercury-containing compounds (‘mercurials’) and thalidomide. All compounds were tested at the maximum non-cytotoxic concentration, while valproic acid and thalidomide were additionally tested over a wide range of concentrations. In total, 242 genes (252 PSs) in the UKK test system and 793 genes (1092 PSs) in the UKN1 test system were deregulated by the 12 test compounds. We identified sets of ‘diagnostic genes’ appropriate for the identification of the influence of HDACis or mercurials. Test compounds that interfered with the expression of developmental genes usually antagonized their spontaneous development, meaning that up-regulated developmental genes were suppressed and developmental genes whose expression normally decreases were induced. The fraction of compromised developmental genes varied widely between the test compounds, and it reached up to 60 %. To quantitatively describe disturbed development on a genome-wide basis, we recommend a concept of two indices, ‘developmental potency’ (*D*_p_) and ‘developmental index’ (*D*_i_), whereby *D*_p_ is the fraction of all developmental genes that are up- or down-regulated by a test compound, and *D*_i_ is the ratio of overrepresentation of developmental genes among all genes deregulated by a test compound. The use of *D*_i_ makes hazard identification more sensitive because some compounds compromise the expression of only a relatively small number of genes but have a high propensity to deregulate developmental genes specifically, resulting in a low *D*_p_ but a high *D*_i_. In conclusion, the concept based on the indices *D*_p_ and *D*_i_ offers the possibility to quantitatively express the propensity of test compounds to interfere with normal development.

**Electronic supplementary material:**

The online version of this article (doi:10.1007/s00204-016-1741-8) contains supplementary material, which is available to authorized users.

## Introduction

Developmental toxicity testing represents a particularly challenging field of toxicology because the currently applied animal tests, such as second-generation reproduction or developmental neurotoxicity studies, are cost as well as labour intensive and require high numbers of animals (Adler et al. 2011; Forsby and Blaauboer 2007; Leist et al. 2008, 2013). In addition, developmental toxicity animal studies are not entirely relevant to the human situation. Therefore, the currently available testing capacities for studying the high number of developmental toxicants are not sufficient (Hengstler et al. 2006; Reif 2014a; Zimmer et al. 2014). A relatively high fraction of almost 20 % of newly approved drugs in Europe were reported to have post-approval issues, and between 2009 and 2011, five drugs had to be withdrawn from the market (Mol et al. 2013). Moreover, the chemical industry has been confronted with the European regulation on Registration, Evaluation, Authorisation and Restriction of Chemicals (REACH) initiative to provide more detailed toxicological data (Hengstler et al. 2006). Therefore, large efforts have been made to develop human stem cell-based in vitro test systems (Hengstler et al. 2006; Meganathan et al. 2015; Shinde et al. 2015; Weng et al. 2014). These in vitro systems recapitulate the critical phases of development, during which they are exposed to test compounds (Krug et al. 2013). Meanwhile, these systems have been applied in numerous studies to identify and characterize developmental toxicants (Balmer et al. 2014; Meganathan et al. 2012, 2015; Sisnaiske et al. 2014; Zimmer et al. 2014). Genome-wide expression studies have been performed to classify developmental toxicants (Rempel et al. 2015), and an eight-gene classifier has been shown to distinguish compounds acting as histone deacetylase inhibitors (HDACis) from a heterogeneous group of ‘mercurials’. Moreover, concentration-dependent, genome-wide expression studies in such stem cell-based systems have revealed concentration progression principles that allow the differentiation of (1) tolerated concentrations, where no gene expression changes are induced, (2) teratogenic concentrations ranges, where critical developmental genes are deregulated but no cytotoxicity occurs and (3) cytotoxic concentrations (Waldmann et al. 2014).

**Fig. 1:**
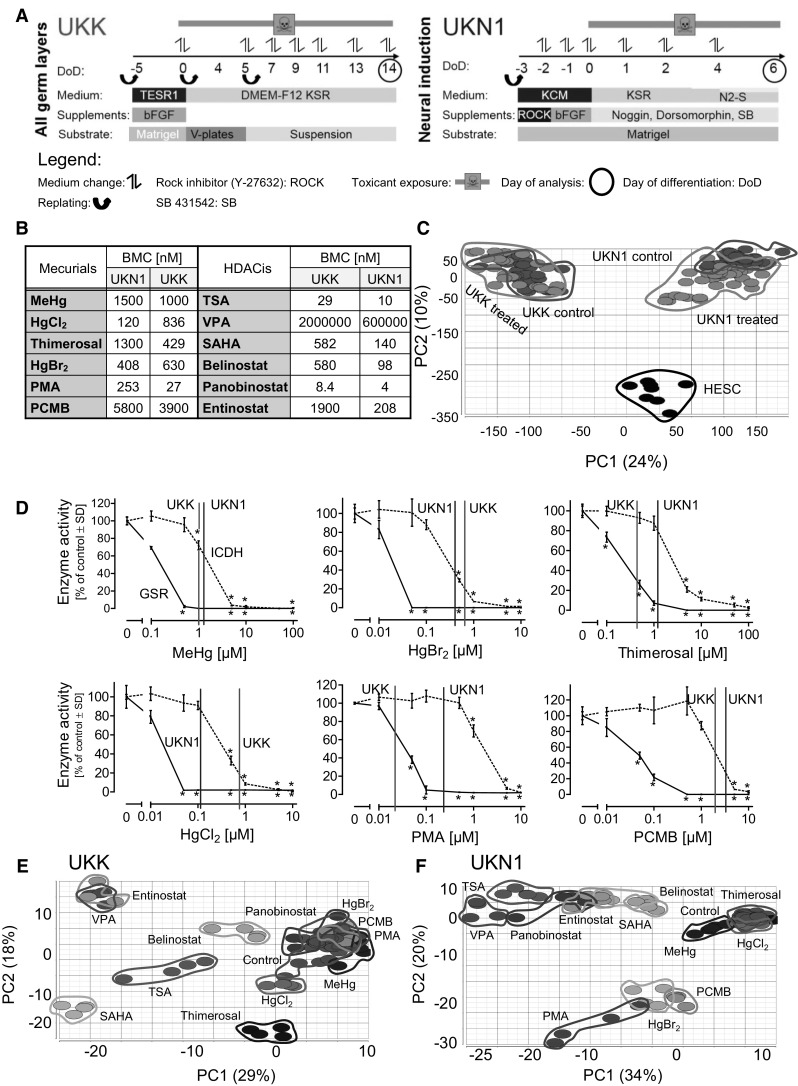
Data structure of transcriptome changes triggered by histone deacetylase inhibitors (HDACis) and mercurials in two human stem cell systems differentiating towards all three germ layers (UKK) and neuroectoderms (UKN1). **a** Stem cells were either differentiated towards all three germ layers (UKK) for 14 days (DoD 14) or towards neuroectoderms (UKN1) over 6 days of differentiation (DoD 6), as indicated. **b** The highest non-cytotoxic concentration, corresponding to EC10, of all test compounds was determined in a viability assay. This ‘benchmark concentration’ (BMC) was used for obtaining transcriptome data of HDACis and mercurial exposure. The BMCs were calculated based on the concentration–response curves of three independent experiments. **c** The data structure of all transcriptome data sets was dimensionality-reduced and presented in the form of a 2D principle component analysis (PCA) diagram. The PCA illustrates a relatively large distance between human embryonic stem cells (hESCs) and differentiated cells at DoD 14 in the UKK system (UKK control) and at DoD 6 in the UKN1 system (UKN1 control). **d** Isocitrate dehydrogenase (ICDH) was incubated for 20 min with mercurials at the indicated concentrations. Isocitrate and NADP^+^ were added to determine the ICDH activity photometrically by measuring the reduction of NADP^+^ to NADPH. The ICDH activity is represented as a percentage relative to untreated control enzyme (*dashed line*). Glutathione reductase (GSR) was incubated for 20 min with the respective mercurials at the indicated concentrations. GSR activity was determined photometrically and is represented as a percentage relative to untreated control enzyme (*solid line*). The BMCs of the respective mercurials (used in this study for microarray analysis) are indicated by a *red line* (UKK) and a *blue line* (UKN1); data are shown as the mean ± SD; *n* = 3. **e**, **f** PCA analysis (using the 50 most regulated genes, defined by the lowest FDR-corrected *p* value) was performed separately for the two systems, including the 12 toxicants (*n* = 4) plus the untreated control (*n* ≥ 8) investigated in them (colour figure online)

A specific feature of stem cell-based developmental in vitro tests is that they represent dynamic systems. During the test period, when stem cells differentiate, for example, to cells of the three germ layers, some hundreds of genes are up- or down-regulated (‘developmental genes’), while expression of the majority of the approximately 22.000 genes of human cells remains unaltered (‘static genes’). Although much progress has been achieved in the field of developmental in vitro tests in the past decade (Reif 2014b; Stober 2014), a comprehensive analysis differentiating the influence of chemicals on ‘developmental’ versus ‘static’ genes and their role in developmental toxicity in humans has not yet been performed. To achieve more progress in this field, we used two recently introduced in vitro systems of human developmental toxicity: the UKK (Universitätsklinikum Köln) test system, which recapitulates the development of human embryonic stem cells (hESCs), or alternatively, of other pluripotent stem cells (hPSCs), to the three germ layers and their derivatives during a 14-day differentiation process, and the UKN1 (University of Konstanz) test system, which represents a 6-day process of hESCs/hPSCs differentiating into neural precursors (Fig. 1a; Krug et al. 2013). We focused on genome-wide expression data of six ‘mercurials’, a relatively heterogeneously acting group of chemicals containing mercury in their molecules, and we compared their effects to those of six histone deacetylase inhibitors, representing a relatively homogeneous group of compounds with a similar mechanism of action. We report that the influence on developmental as well as static genes is highly compound specific. Moreover, different developmental processes, such as the induction of the germ layer or different somatic cells, or specific neural induction, show differential susceptibility to individual test compounds. In the present study, we introduce two basic developmental indices to characterize the potency of developmental toxicants: (1) developmental potency (*D*_p_), which represents the fraction of developmental genes among all genes whose expression is influenced by a test compound, and (2) developmental index (*D*_i_), which describes the degree to which developmental genes are overrepresented among all genes deregulated by a test compound. These parameters can easily and precisely be determined and represent a sound starting point for a more detailed understanding of the disturbed development of differentiating stem cells. Overall, the tests are based on stem cell and omics technologies and have a high predictive potency for discriminating between general and developmental toxicity. Accordingly, we have named the UKK test system as the STOP-Tox_ukk_ test and the UKN1 system as the STOP-Tox_ukn_ test (STOP, **S**tem cell-based **T**eratogenic **O**mics **P**rediction).

## Materials and methods

### Materials

Gelatin, putrescine, selenium, progesterone, apotransferrin, glucose and insulin were obtained from Sigma (Steinheim, Germany). Accutase was obtained from PAA (Pasching, Austria). FGF-2 (basic fibroblast growth factor), noggin and sonic hedgehog were obtained from R&D Systems (Minneapolis, MN, USA). Y-27632, SB-43154 and dorsomorphin dihydrochloride were obtained from Tocris Bioscience (Bristol, UK). Matrigel^TM^ was obtained from BD Biosciences (Massachusetts, USA). All cell culture reagents were obtained from Gibco/Invitrogen (Darmstadt, Germany), unless otherwise specified. The following chemicals (HDACis and mercurials) were obtained from Sigma unless otherwise specified. The vehicles used are also mentioned with the compounds: (HDACis) valproic acid (VPA, P4543; water); trichostatin (TSA, T1952; DMSO); vorinostat (SAHA, SML 0061; DMSO); belinostat (PXD101, S1085, Selleckchem; DMSO); panobinostat (LBH589, S1030, Selleckchem; DMSO); entinostat (MS-275, Cay-13284-25; Biomol; DMSO); (mercurials) methylmercury (MeHg, 442,534; 10 % ethanol); thimerosal (THM, T4687; water); mercury(II)chloride (HgCl_2_, 203,777; water); mercury(II)bromide (HgBr_2_, 437859, water); 4-chloromercuribenzoic acid (PCMB, C5913-5G; water); and phenylmercuric acetate (PMA, P27127-25G).

### Human embryonic stem cells

The human pluripotent stem cell line H9 (Thomson et al. 1998) was obtained as a WA09 line from WiCell (Madison, WI, USA). The importation of the cells and the subsequent experiments were authorized (Robert Koch Institute, Berlin, Germany) under licence # 170-79-1-4-27 for the UKN1 system and licence # 1710-79-1-4-34 for the UKK system. H9 cells were cultured on irradiated mouse embryonic fibroblasts (MEFs) in H9 culture medium, as previously described (Jagtap et al. 2011; Krug et al. 2013; Shinde et al. 2015).

### Random differentiation of hESCs to germ layer cell types and their derivatives (UKK test system)

To remove MEFs, H9 cells from the maintenance culture were transferred on hESC-qualified matrix (BD Biosciences, California, USA)-coated, 60-mm tissue culture plates (Nunc, Langenselbold, Germany) in TESR1 medium (StemCell Technologies) and were maintained for 5 days prior to differentiation. The random differentiation into embryoid bodies (EBs) representing multiple lineages was performed as described previously (Meganathan et al. 2012). In brief, cell clumps were obtained by cutting and scraping the cells with a passage tool (StemPro EZPassage™ Disposable, Invitrogen) and a cell scraper. On day 0, 80 clumps were seeded in each well of a pluronic-coated, *v*-bottom plate in 100 µl of random differentiation (RD) medium (DMEM-F12 medium with 20 % KO serum replacement, 1 % non-essential amino acids, penicillin (100 units/ml), streptomycin (100 µg/ml) and 0.1 mM β-mercaptoethanol) containing chemical or vehicle, and the plate was then incubated (37 °C, 5 % CO_2_) for 4 days. The EBs were collected on day 4 and were transferred onto a 100-mm bacteriological plate in 15 ml of RD medium containing the chemical or vehicle. The medium was replenished every alternate day until day 14 of differentiation.

### Neuroepithelial differentiation (UKN1 test system)

H9 cells were cultured according to standard protocols and were differentiated into neuroepithelial progenitors (NEPs) as previously described (Balmer et al. 2014; Krug et al. 2013; Shinde et al. 2015) and as shown in Fig. 1. The differentiation of the H9 cells towards NEPs was based on a dual SMAD inhibition (Chambers et al. 2009) using a combination of 35 µM noggin and 600 nM dorsomorphin together with 10 µM SB-431642. This was used to prevent BMP and TGF signalling and thus achieve a highly selective neuroectodermal lineage commitment. Cells were handled and manipulated as previously described in the supplemental methods (Balmer et al. 2014).

### Benchmark concentration (BMC) determination

The BMC is defined here as the concentration at which at least 90 % of the cells survive (BMC10). For both test systems, the BMC10 was determined for six HDACis and six mercurial compounds by performing resazurin cell viability assays exactly as previously described (Krug et al. 2013; Stiegler et al. 2011). In the UKK system, the H9 cells were differentiated as described above and were treated from DoD 4 to DoD 14. In the UKN1 test system, the cells were treated from DoD 0 to DoD 6. Both test systems were treated during the indicated time periods with concentrations ranging from non-cytotoxic to cytotoxic. The analysis was performed on DoD 14 (UKK) and DoD 6 (UKN1). The experiments have been performed with five technical and three biological replicates. On the day of analysis, cells were incubated with 10 µg/ml resazurin for 1–1.5 h at 37 °C and 5 % CO_2_. To determine the background fluorescence of resazurin itself, a control with only resazurin in medium was included. Resorufin was measured at a wavelength of 560Ex/590Em with a fluorescence reader. The mean background fluorescence of resazurin was subtracted from all experimental data. Further data processing to identify BMC of chemicals was done as mentioned below. Data from each experiment were normalized to their respective vehicle controls (set as 100 %). The data were then displayed in semi-logarithmic plots. Data points were connected by a nonlinear regression sigmoidal dose–response curve fit. These curves were averaged, and BMC10 was then determined graphically as the data point on the average curve corresponding to 90 % viability value, or as the last real data point left of this value. The BMC10 concentrations of the compounds were considered for further Affymetrix-based studies with UKK and UKN1 protocol.

### Affymetrix-based study details

For Affymetrix-based study, six HDACis and six mercurial compounds were exposed at BMC10 concentrations from DoD 0 to DoD14 or from DoD 0 to DoD6 in UKK or UKN1 test system, respectively, along with the respective vehicle controls. The samples were collected on DoD 14 or DoD 6 for RNA isolation. The samples from four biological replicates were used for further processing.

### Affymetrix DNA microarray analysis

On DoD 14 (UKK) or DoD 6 (UKN1), medium was removed, and the cells were lysed in RNA protect solution (Qiagen). Affymetrix chip-based DNA microarray analysis (Human Genome U133 plus 2.0 arrays) was performed exactly as previously described (Krug et al. 2013; Meganathan et al. 2015). Briefly, total RNA was isolated using TRIzol and chloroform (Sigma, Steinheim, Germany) and purified with miRNeasy mini kit (Qiagen, Hilden, Germany). The quantification and quality control measurements were done using Nanodrop spectrophotometer (ND-1000, Thermo Fisher, Langenselbold, Germany). For microarray labelling 100 ng total RNA was taken as a starting material, and after amplification 12.5 µg amplified RNA was hybridized on Affymetrix Human Genome U133 Plus 2.0 arrays (Affymetrix, Santa Clara, CA, USA). For washing and staining Affymetrix HWS kit and Genechip Fluidics Station-450 were used according to the manufacturer’s instructions. After staining arrays were scanned with Affymetrix Gene-Chip Scanner-3000-7G and Affymetrix GCOS software has been used for quality control analysis. The corresponding raw CEL files of the Affymetrix chips are publicly available under GEO accession number GSE71127.

### Biostatistics

Batch effects, non-biological experimental variation, is a commonly observed phenomenon in the area of microarray studies. They occur due to experiments that cannot be conducted all at once, for various reasons. It was important to eliminate batch effects as otherwise relevant expression changes may remain undetected. We applied the ComBat algorithm (Johnson et al. 2007) that allowed adjusting for batch effects in datasets where the batch covariate was known. It used a nonparametric, empirical Bayes approach for the estimation of an additive and a multiplicative batch effect. First, we used extrapolation strategy (RMA +) (Harbron et al. 2007) for background correction, log2 transformation and quantile normalization. Then the data were standardized with respect to mean gene expression and treatment effect. Then, the batch effects were estimated and eliminated from the standardized data by subtracting the additive effect and dividing by the multiplicative effect. Finally, the data were back-transformed, i.e. mean gene expression and treatment effect were added. Further statistical data analysis and visualization performed by uploading ComBat-corrected files into the Partek Genomics Suite (PGS) version 6.6 software (Partek, St. Louis, MO, USA). The normalized probe sets (PSs) were used for the generation of a principal component analysis (PCA) and one-way ANOVA model that was used to identify the differentially regulated transcripts with changes of at least 1.5-fold (absolute fold change, *p* value ≤0.05), with Benjamini and Hochberg FDR corrections. The first 50 transcripts deregulated by each toxicant were filtered based on *p* value, and signals were normalized by z-score and clustered using a hierarchal cluster analysis (complete linkage method). The commonly deregulated transcripts were obtained using a Venn diagram overlap analysis (PGS). Online free software such as g:Profiler and the Database for Annotation, Visualisation and Integrated Discovery (DAVID) were used for functional annotation and gene ontology (GO) clustering of differentially expressed transcripts (*p* ≤ 0.05).

#### Construction of a transcription factor network

The TF network was constructed as previously described (Rempel et al. 2015). Briefly, we downloaded raw data for the microarray samples referenced in the manually curated CellNet tissue atlas (Cahan et al. 2014) and combined them with data from the UKN1 and UKK test systems (Balmer et al. 2014; Cahan et al. 2014; Krug et al. 2013; Waldmann et al. 2014). To obtain the expression matrix, the samples were normalized together using RMA implemented in the R package oligo. The co-expression network was constructed in two steps using functions from the parmigene package for R. First, the mutual information matrix was computed by applying the function knnmi.all with parameter *k* = 9, chosen based on an unpublished benchmarking, on the expression matrix. Then, we applied the clr function from the parmigene package, which implements the CLR algorithm. The co-expression network was subsequently restricted to genes annotated as transcription factors (TFs) in the Animal Transcription Factor Database (AnimalTFDB, [http://www.bioguo.org/AnimalTFDB/index.php]). The overlap of the genes detected by the Affymetrix array and the AnimalTFDB was 1300 genes. Links were drawn only for pairs of TFs with a score in the top 0.1 % of all co-expression scores. This yielded 1690 predicted interactions between 847 TFs. Nodes were arranged in the network according to the Fruchterman and Reingold’s force-directed placement algorithm provided by the R package sna with the area parameter = 10^9^.

#### Representation of mercurials or HDACis consensus genes on the TF network

Communities of network nodes were determined by the fast greedy community function of the R package igraph. Only the top 18 largest communities were analysed for the enrichment of GO biological process annotations, as the others contained less than six genes. The enrichment analysis was performed with the R package topGO using the classic method and the Fisher’s test statistic. We selected representative terms for each community from the top enriched terms with an unadjusted *p* value <0.05. For spontaneous differentiation and regulation by compounds, TFs in the network were marked red (blue) if a probe set mapping to this TF was up-regulated (down-regulated) under the respective condition. The mapping of PSs to the Ensembl gene ids and gene symbols was determined using the BioConductor package hgu133plus2.db. Only PSs that could be mapped to a gene symbol were taken into account. TFs for which PSs mapping to them were inconsistently regulated were removed from the analysis.

#### Glutathione reductase (GSR) and isocitric dehydrogenase (ICDH) activity assays

ICDH (porcine, Sigma, I-2002) (10 µg/200 µl) in a Tris(hydroxymethyl)-aminomethane (Tris)-buffer (20 mM) containing MnSO_4_ (2 mM), pH 7.4, was incubated with the compounds to be tested at 37 °C for 20 min. ICDH activity was determined by the addition of isocitrate (4 mM) and NADP^+^ (0.1 mM). The enzymatic reduction of NADP^+^ to NADPH was monitored using photospectroscopy at 340 nm over the course of 15 min at 1-min intervals and 37 °C. The enzymatic activity was determined from the slope of the absorbance increase over time. All data were normalized to the activity of untreated enzyme (i.e. free of toxicant). GSR (human, Sigma G-9297) (10 µg/200 µl) was incubated in sodium phosphate buffer (100 mM), pH 7.5, containing ethylenediaminetetraacetic acid (EDTA; 1 mM) and the compounds to be tested for 20 min at 37 °C. To assess GSR activity, oxidized glutathione (GSSG) (5 µM), NADPH (0.4 mM) and 5,5′-dithiobis(2-nitrobenzoic acid) (DTNB) (all from Sigma) were added, and the reaction was monitored by absorbance measurements at 405 nm (37 °C) at 1-min intervals over the course of 15 min. The enzymatic activity was determined from the slope of the absorbance increase over time. All data were normalized to the activity of untreated enzyme (i.e. free of toxicant).

#### Identification of consensus genes

A gene was defined as significantly deregulated by a specific compound if at least one annotated probe set was significantly deregulated (absolute fold change >1.5 and FDR-corrected *p* value <0.05). A gene was defined as a ‘consensus’ gene if it was significantly up- or down-regulated by as many compounds of same class as possible (i.e. mercurial or HDACi).

#### Identification of diagnostic genes

A ranking approach was performed to identify PSs that fulfilled the following criteria: (1) deregulation occurred from as many compounds of the same class as possible (i.e. HDACi or mercurial); (2) PSs with higher fold changes compared with those of the controls were preferentially considered; (3) only the developmental genes were considered; (4) PSs were only considered when the test compounds antagonized the spontaneous development, i.e. when up-regulated developmental genes were suppressed or down-regulated developmental genes were induced; (5) only PSs with baseline expression values >6 (log2 scale) at day 0 or at the day of differentiation (day 14 in the UKK system or day 6 in the UKN1 system) were considered (the number of PSs passing this criteria are shown in Suppl. Fig. S5A &B, the cut-off value has been selected based on the frequency distribution curves provided in Suppl. Fig. S5C & D); and (6) PSs were only considered when they could be assigned to genes whose function is basically understood.

## Results

### Structure of developmental genes in differentiating stem cells

In the present study, two stem cell-based test systems were used, the UKK system, where hESCs/hPSCs differentiate into cells of the three germ layers and their derivatives, and the UKN1 system, which recapitulates differentiation into early neural precursor cells (Fig. 1a). Genome-wide gene expression profiles were established from the undifferentiated hESCs and after 14 (UKK) and 6 days (UKN1) of differentiation. Additionally, hESCs of both in vitro systems were exposed to 12 test compounds at benchmark concentrations of low cytotoxicity during the differentiation periods. Among them, six mercurials and six HDACis were applied (Fig. 1b, c). All compounds were tested at their maximum non-cytotoxic concentrations, i.e. at the benchmark concentration (BMC10) resulting in a maximal viability reduction of 10 %. This biological-anchoring point has been previously found to be well suited for transcriptome analysis (Waldmann et al. 2014). Moreover, it is also well correlated with known pharmacological and toxicological molecular properties of the test compounds. For instance, the concentrations of the HDACis were all within the range known to be required for half-maximal enzyme inhibition in biochemical assays (Rempel et al. 2015). The concentrations of the mercurials were all in a similar range with respect to the inhibition of thiol-containing, redox-sensitive enzymes. Glutathione reductase (GSR) or isocitrate dehydrogenase (ICDH) were chosen as target enzymes for this rough bioequivalence test, and the BMC10 test concentrations all led to partial enzyme inhibition (Fig. 1d). Notably, none of the HDACis affected the enzyme activities at the concentrations tested here. To obtain an overview over the genome-wide data, principle component analysis (PCA) plots were established. The PCA illustrates a relatively large distance between hESCs and the cells resulting after the 14-day (UKK) and 6-day (UKN1) differentiation processes (Fig. 1c). Compared with these large distances, the influence of the test compounds appears comparatively small (Fig. 1c). However, when the PSs for the PCA analysis were limited to the 50 most regulated (defined by the lowest FDR-corrected *p* values) of each test substance, most compounds cluster distinctly from the controls, whereby the differentiation between compound-exposed samples and their controls appears more separated in data from the UKN1 system than in data from the UKK system (Fig. 1e, f).

**Fig. 2:**
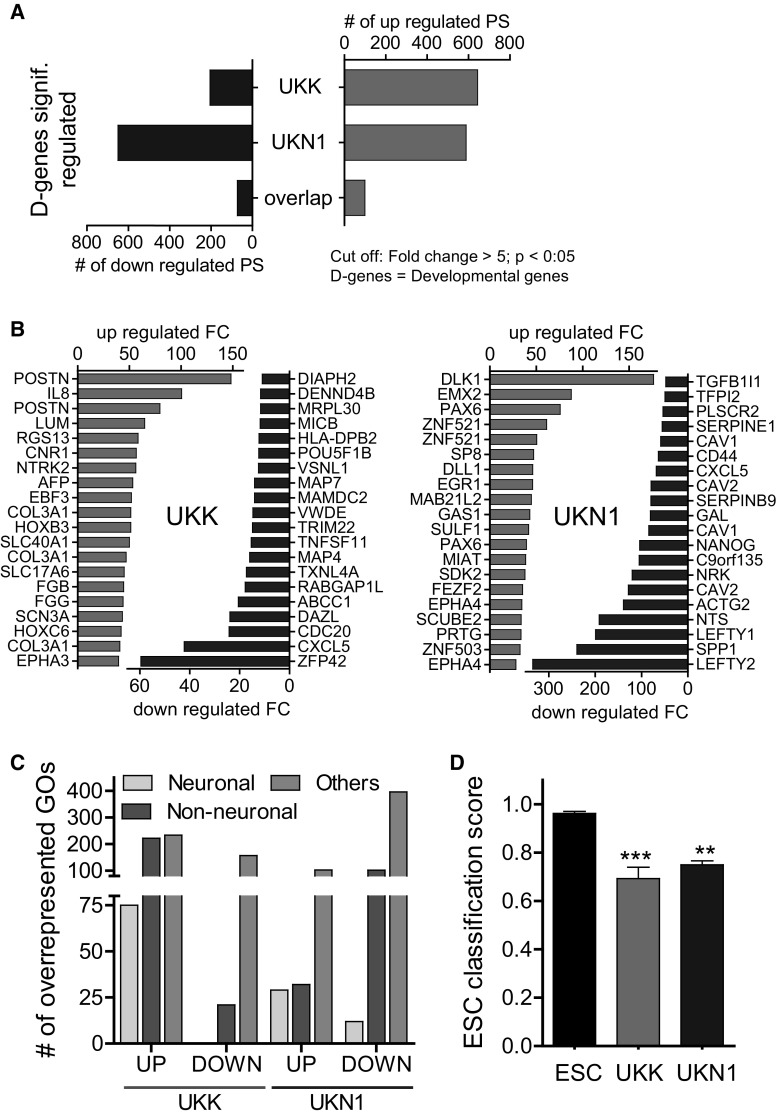
Characterization of the two test systems, UKK (three germ layer) and UKN1 (neuroectoderm), by transcriptome analysis. Human ESCs were differentiated as indicated in Fig. 1a and were used for whole-transcriptome analysis. **a** Number of up-(*red*) and down-(*blue*) regulated PSs at DoD 14 in the UKK system and at DoD 6 in the UKN1 system (D-genes). The overlap of D-genes in the UKK and UKN1 test systems up- and down-regulated by ≥fivefold is shown (detailed data are shown in supplemental materials). **b** Top 20 significantly up-(*red*) and down-(*blue*) regulated genes for the UKK system (*left*) and the UKN1 system (*right*). **c** The gene ontology (GO) categories belonging to biological processes overrepresented amongst up- and down-regulated genes (*p* < 0.05) were subcategorized into three classes: ‘neuronal development’, ‘non-neuronal development’, and ‘others’. The *number* of these overrepresented GO categories up- and down-regulated in the UKK and UKN1 systems are shown. **d** CellNet analysis shows the ESC classification score for ESCs and differentiated cells at DoD 14 in the UKK system and at DoD 6 in the UKN1 system (detailed data for the tissue classification scores are shown in the supplemental materials) (colour figure online)

Subsequently, we addressed genes whose expression alters spontaneously during the differentiation of stem cells. Genes that are up- or down-regulated during differentiation into the germ layers and their derivatives (UKK) or into neuronal precursor cells (UKN1) will be further referred to here as ‘developmental genes’. There were no major differences in the number of developmental up-regulated genes in the UKK and UKN1 systems, but fewer genes were down-regulated in the UKK system (Fig. 2a). The overlap of developmental genes between the UKK und UKN1 systems was relatively small (Suppl. Table 1). A relatively high fraction of developmental genes showed high fold changes (Suppl. Table 1). The number of PSs up-regulated by at least fivefold (absolute value, *p* < 0.05; FDR-adjusted) was 545 specifically in the UKK system, 489 specifically in the UKN1 system, and 99 PSs were up-regulated in both systems. The corresponding numbers of down-regulated PSs were 132, 577 and 73, respectively. Although there was little overlap of the strongest up- or down-regulated developmental genes between the UKK and UKN1 systems (Fig. 2b), the gene ontology (GO) categories of both systems was similar, with a high fraction of development-associated motives (Table 1). Further differentiation of the development-associated GO groups into neuronal and non-neuronal development illustrated that a higher number of neuronal development-associated GO groups was down-regulated in the UKN1 system compared with that in the UKK system (Fig. 2c; Suppl. Table 2). A similar conclusion was obtained by the analysis of PSs associated with nervous system development. No nervous system development-associated PSs were significantly down-regulated in the UKK system compared with the 139 that were in the UKN1 system (Suppl. Fig. S1). To obtain a deeper understanding of the differentiation process in the UKK and UKN1 systems, we performed a gene regulatory network analysis (CellNet), which quantifies how closely engineered cell populations resemble specific human cell types (Cahan et al. 2014; Godoy et al. 2015). CellNet showed a decrease in the ESC score after the differentiation period in both systems, UKK and UKN1 (Fig. 2d). However, the increase in tissue classification scores was only small (Suppl. Fig. S2), which was expected because the cells generated in the UKK and UKN1 systems are known to represent precursor and not yet mature cell (Krug et al. 2013). While the UKN1 system reached the highest tissue classification score for neuronal cells, the UKK system scored higher for a broader set of cell types, including lung, skin, liver, kidney and heart cells as well as fibroblasts (Suppl. Fig. S2). The results of the regulatory network analysis are in agreement with the concept that the UKK differentiation protocol allows the development of all three germ layers and their derivatives, while development within the UKN1 system is guided towards neuronal precursors, and the development of further cell types is suppressed. Although the differentiation protocols of the UKK and UKN1 systems did not result in qualitative (i.e. all or nothing) differences between the test systems, the obtained quantitative differences are robust, considering the relatively small error margins and non-overlapping scores of the independent experiments (Suppl. Fig. S2).

**Table 1:** Top 10 gene ontology categories overrepresented amongst up- and down-regulated probe sets during differentiation

Regulation	System	Neuronal	*p* value	Non-neuronal	*p* value
UP ↑	UKK	Nervous system development	5.1E−56	Anatomical structure dev.	2.3E−45
		Neurogenesis	5.5E−42	Developmental process	8.0E−44
		Generation of neurons	1.7E−41	Tissue development	5.1E−23
		Neuron differentiation	6.2E−37	Epithelium development	1.0E−16
		Central nervous system development	6.3E−32	Muscle tissue development	1.7E−14
		Brain development	3.5E−26	Striated muscle tissue dev.	2.6E−14
		Neuron development	6.2E−26	Connective tissue development	7.5E−13
		Regulation of nervous system dev.	1.8E−24	Mesoderm development	1.2E−03
		Forebrain development	5.3E−23	Endothelium development	1.3E−03
		Neuron projection development	3.0E−21	Palate development	2.9E−03
	UKN1	Nervous system development	6.2E−19	Single-organism dev. process	5.1E−12
		Generation of neurons	5.1E−13	Head development	2.4E−10
		Central nervous system development	1.0E−12	Regulation of dev. process	9.1E−10
		Neurogenesis	1.8E−12	Anatomical structure dev.	2.5E−09
		Forebrain development	5.8E−11	Multicellular organismal dev.	1.4E−08
		Neuron differentiation	6.4E−11	Anatomical structure morphogenesis	1.4E−08
		Brain development	3.1E−10	System development	2.1E−07
		Regulation of nervous system dev.	7.5E−08	Cell differentiation	9.1E−07
		Negative reg. of nervous system dev.	4.1E−07	Negative regulation of cell dev.	9.8E−07
		Negative reg. of neurogenesis	1.5E−06	Regulation of organismal dev.	1.4E−06
DOWN↓	UKK			Anatomical structure development	2.7E−12
				Single-organism dev. process	1.3E−11
				Multicellular organismal development	1.6E−11
				System development	2.8E−09
				Cell differentiation	4.2E−09
				Organ development	6.4E−08
				Anatomical structure morphogenesis	2.6E−07
				Tissue development	5.0E−07
				Anatomical structure formation	4.1E−06
				Circulatory system development	2.6E−05
	UKN1	Neuron projection dev.	5.2E−07	Anatomical structure morphogenesis	8.4E−26
		Neuron projection morphogenesis	3.5E−06	System development	1.3E−24
		Neuron development	1.8E−05	Tissue development	1.7E−24
		Nervous system development	2.1E−05	Anatomical structure development	1.1E−23
		Neurogenesis	3.1E−05	Developmental process	5.8E−21
		Generation of neurons	5.6E−05	Multicellular organismal development	1.5E−20
		Neuron differentiation	1.6E−04	Single-organism dev. process	3.8E−19
		Axon development	1.9E−04	Circulatory system development	7.4E−19
		Cell morphogenesis in neuron diff.	3.7E−04	Cardiovascular system development	7.4E−19
		Axonogenesis	4.9E−04	Organ development	2.7E−18

**Fig. 3:**
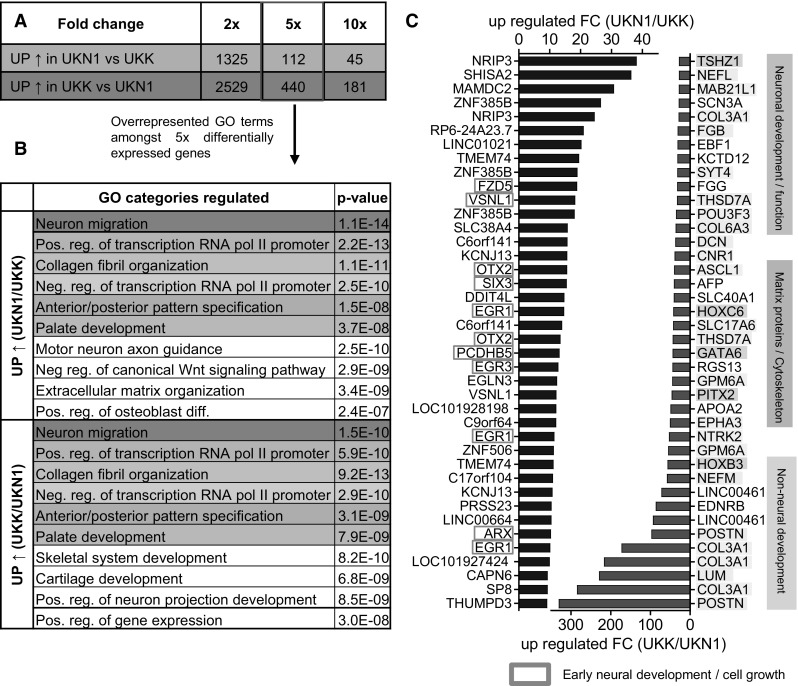
Direct comparison of the end-stage cells in the UKK and UKN1 test systems by transcriptome analysis. Human ESCs were differentiated as indicated in Fig. 1a and were used for whole-transcriptome analysis. **a** The *number* of differentially expressed PSs (fold change ≥2-/5-/10-fold, FDR-corrected *p* value <0.05) in the UKN1 system compared with the UKK system. **b** The top 10 overrepresented GO terms amongst ≥fivefold differentially expressed genes are shown. The top 10 GO terms were sorted by similarity, and the *colours* highlight identical GO terms. **c** The top 40 significantly up-regulated (*blue*) PSs in the UKN1 system compared with the UKK system and up-regulated (*red*) PSs in the UKK system compared with the UKN1 system, sorted by fold expression. The PSs were marked according their role in superordinate cell biological processes: ‘early neural development’ (*blue encircled*), ‘neuronal development/function (*yellow*), ‘extracellular matrix proteins/cytoskeleton/cell growth (*cyan*) and non-neural development (*pink*) (colour figure online)

An overview over the most differential genes, GO categories and the most deregulated genes between both systems is given in Fig. 3. Over 3800 PSs were differentially expressed (absolute twofold difference) in the end stage of the UKN1 and UKK cell systems. When the analysis was limited to differentially expressed genes (DEGs) that were at least fivefold difference (or tenfold, absolute values, for the UKK system), 112 (45) PSs were found to be more highly expressed in the UKN1 system, and 440 (181) were more highly expressed in the UKK system (Fig. 3a). An analysis of overrepresented GOs amongst the DEGs (5-fold difference) showed a highly significant enrichment of GO terms (*p* < 10E−10), such as ‘neuron migration’, ‘collagen fibril organization’ and ‘anterior/posterior pattern specification’. However, many of the terms were overrepresented both amongst DEGs that were high in the UKK system and those that were high in the UKN1 system (Fig. 3b). A potential explanation for this unexpected, but interesting, result is that genes from these domains are dynamically regulated in both systems and are thus most likely to show differential expression from system to system. It has been previously demonstrated that the analysis of overrepresented GO terms is often not a very sensitive method to unravel complex biological regulations (Kuegler et al. 2010; Zimmer et al. 2011). For this reason, the most strongly differentially expressed genes were identified and scrutinized individually (Fig. 3c). Amongst the top 40 UKN1-specific genes, a larger group was found to be involved in early neural development and cell growth, consistent with the very early developmental status of cells in this test system. Amongst the UKK-specific genes, three conspicuous groups were identified: ‘neuronal development/function’, ‘extracellular matrix proteins/cytoskeleton’, and ‘non-neural development’. This observation is well in line with the UKK system allowing for the maturation of neural cells towards neurons, with the parallel development of non-neuronal cells, and with the more tissue-like structure of the 3-dimensional aggregates of that test system (Fig. 3c).

### Interference of chemicals with stem cell differentiation

**Fig. 4:**
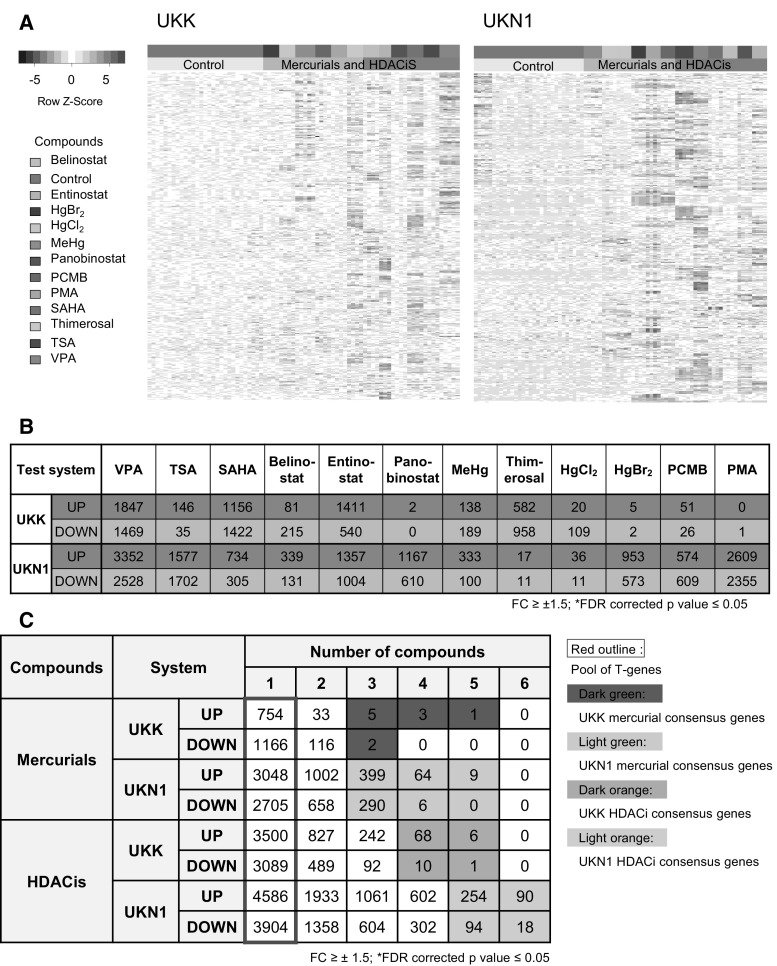
Characterization of transcriptional changes induced by HDACis and mercurials, and identification of toxicant class consensus genes for the UKK and UKN1 systems. Differentiating cells were treated with mercurials and HDACis as indicated in Fig. 1a and were used for transcriptome analysis. **a** The 50 most significant transcripts de-regulated by each toxicant were used for hierarchical cluster analysis (complete linkage method). The results are represented as a heat map, with each column representing one experiment, each row indicating data for one probe set, and the colour of each cell indicating the row-wise *z*-score of gene expression levels (*blue indicates low* and *red indicates high*). **b** The number of differentially expressed PSs (fold change ≥±1.5, FDR-corrected *p* value < 0.05) after exposure to toxicants compared with those of untreated controls (detailed data are shown in supplemental materials). **c** Amongst the differentially expressed PSs, the number of PSs that were up- and down-regulated by exactly 1, 2, 3, 4, 5 or 6 mercurials or HDACis in the UKK and UKN1 systems were counted. The columns in the cross table indicate how many PSs were up-(or down-) regulated, e.g. by four mercurials. For instance, 64 PSs were up-regulated in the UKN1 system by four mercurials, and 10 PSs were down-regulated in the UKK system by four HDACis. The number of PSs that were influenced by at least one toxicant was referred to as T-genes and is outlined in red (detailed data for the consensus genes are shown in supplemental materials). Mercurial consensus genes were identified in the UKK system (*dark green*, regulated by at least 3 compounds) and the UKN1 system (*light green*, regulated by at least 4 compounds). HDACis consensus genes were identified in the UKK system (*brown*, regulated by at least 4 compounds) and the UKN1 system (*light orange*, regulated by at least 5 compounds) (colour figure online)

**Fig. 5:**
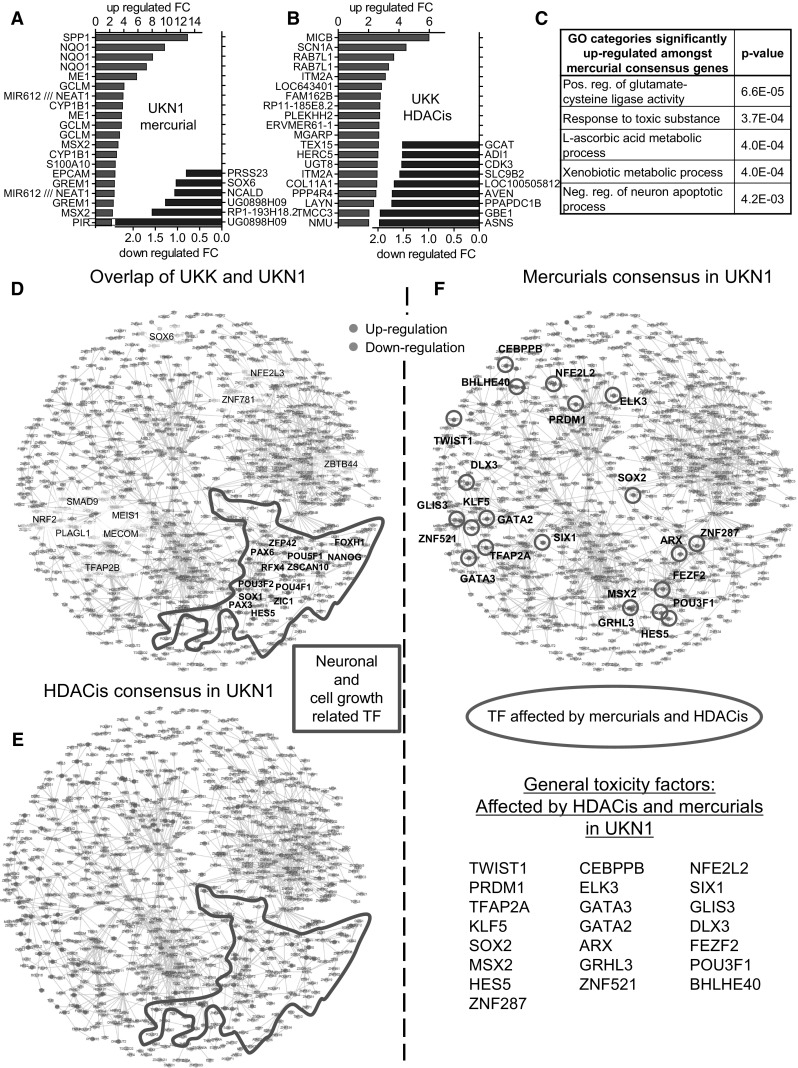
Characterization of HDACi and mercurial consensus genes in the UKK and UKN1 test systems. Human ESCs were differentiated and treated as shown in Fig. 1a and toxicant consensus genes were identified from the transcriptome data as shown in Fig. 4. For each consensus gene, the mean fold change (FC) of all six HDACis or mercurials in each system was calculated and used for further analysis. **a** The top 20 up- and down-regulated mercurial consensus PSs in the UKN1 system (regulated by at least 4 mercurials) are displayed. **b** The top 20 up- and down-regulated HDACi consensus genes in the UKK system (regulated by at least 4 HDACi) are shown. **c** The gene ontology (GO) categories amongst up- and down-regulated mercurial consensus genes in the UKN1 system were identified and sorted by *p* value; the top 5 (lowest *p* values) are displayed. **d** The CellNet database (3297 transcriptome sets from all major tissues) was used to construct a generic human TF network based on statistical co-expression information and graph-theoretical design principles. Each node represents a TF gene, and each edge suggests co-regulation. The edge length is driven by the number of edges on neighbouring nodes. The nodes were placed according to the Fruchterman–Reingold algorithm, and an optimization algorithm that maximized the modularity of the division of the graph into clusters was used to define the clusters. Next, GO term overrepresentation analysis was performed for each cluster to identify its biological role (Rempel et al. 2015). The ‘forebrain development’ and ‘neuronal development’ as well as the ‘cell division’ clusters have been encircled for better visualization. The TFs that were found both amongst the UKK and UKN D-genes (regulated by ≥±5-fold, *p* < 0.05) were selected and highlighted in the TF network (*red indicates up*-*regulation*, *blue indicates down*-*regulation*). **e** All TFs were identified amongst the HDACi consensus genes in the UKN1 system (regulated by at least 3 HDACis) and were highlighted in the TF network. **f** All TFs amongst the mercurial consensus genes in the UKN1 system were identified (regulated by at least 3 mercurials) and were highlighted in the TF network. The mercurial consensus TFs that were also affected by HDACis were *encircled green* and listed below (*red indicates up*-*regulated*, *blue indicates down*-*regulated*) (colour figure online)

Compared with the large gene expression alterations during the differentiation periods of the UKK and UKN1 test systems, the impact of chemicals appears comparatively small in the PCA overview (Fig. 1c). Nevertheless, several chemicals, particularly the HDACis, caused a shift of the exposed samples away from the respective controls (Fig. 1e, f). Cluster analysis based on the 50 most regulated PSs (defined by the lowest FDR-corrected *p* value) by each toxicant (all in cases regulated PS <50) illustrates the separation of solvent controls from the compound-exposed samples (Fig. 4a). The numbers of significantly deregulated PSs (FDR-adjusted) were relatively high for most compounds, usually ranging between 100 and 3000 up- or down-regulated genes (Fig. 4b; Suppl. Table 3). The mercurials HgBr_2_, PCMB and PMA were exceptions, as these caused only small expression alterations in the UKK system. The analysis of the size of significant expression alterations illustrated that much larger fold changes occur during the differentiation process compared with the fold changes induced by chemicals (Suppl. Fig. S3). Analysing the overlap of PSs deregulated by the compounds revealed a consensus signature of 90 up- and 18 down-regulated PSs deregulated by all 6 HDACis in the UKN1 system (Fig. 4c, Suppl. Table 4). For HDACis in the UKK system and for mercurials in both test systems, no consensus PSs for all six test substances could be identified (Fig. 4c). The numbers of the most consensual PSs are indicated by green (mercurials) and orange (HDACis) backgrounds in Fig. 4c, and the corresponding genes are summarized in Fig. 5.

The consensus PSs for HDACis in the UKN1 test system have been characterized previously (Rempel et al. 2015), and the mercurial consensus PSs (deregulated by at least of 3 mercurials) in the UKN1 test system are shown in Fig. 5a. For the UKK test system, no mercury consensus PSs were identified, but there was a clear group of HDACi consensus genes (deregulated by at least of 4 HDACi), and this is shown in Fig. 5b. To study whether consensus genes were derived from specific biological processes triggered by toxicants, overrepresented GO terms were identified amongst the UKN1 mercurial consensus PSs. This analysis indicated the up-regulation of two antioxidant stress response pathways (glutamate–cysteine ligase activity, required for glutathione synthesis; ascorbate metabolism), and two toxicant response pathways. Moreover, there was an indication of the regulation of the anti-apoptotic response in neurons (Fig. 5c).

To further understand the coordinated regulations leading to changes of the consensus genes on a biological system level, disturbances of the transcription factor (TF) network were visualized. For this purpose, we used a previously generated generic TF coexpression network for human cells (Rempel et al. 2015). Within the network, we previously identified groups of three highly connected TFs (communities) that are enriched for GO terms relevant for neural development (Rempel et al. 2015). On this network, we then mapped and compared the TFs changing in the respective test systems (‘developmental TFs’). Some of them were specific for either the UKK or UKN1 system (Suppl Fig. S7). However, there were also TFs regulated in both systems (overlap TFs). A high fraction of these TFs are concentrated in three communities related to neuronal development (56 %). These three communities consist of two communities enriched for neuronal development GO terms and a third community that links the two that contains stem cell factors and cell cycle regulators (Fig. 5d). Subsequently, we identified TFs amongst the toxicant consensus genes and their positioning in the network was visualized (Suppl Fig. S7). For the UKN1 test system, different consensus TFs were visualized. For HDACis, we find that about one-third (32.7 %) of TFs affected by at least three out of six compounds in the UKN1 system mapped to the three communities related to neuronal development (Fig. 5e). For the mercurial consensus (at least 3 out of 5 compounds), we find overall less affected TFs, and also a lower fraction of neuronal development TFs affected (26.5 %) (Fig. 5f). Taken together, the HDACi consensus affected TFs are more strongly concentrated in neuronal development than the mercurial consensus affected TFs. In addition, mercurial consensus TFs mapped relatively broadly across the TF network (Fig. 5f). Most of the TFs affected by mercurials were also affected by HDACis, independent of the location in the network. Such TFs are promising candidates for master regulators that are generally affected by various toxicants in the test systems (Fig. 5f). They may be used in the future as biomarkers, and they may also lead to further mechanistic studies with increased sensitivity for examining mechanisms of toxicity.

**Fig. 6:**
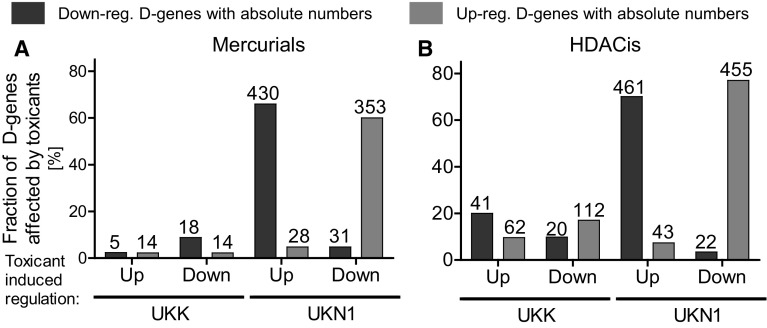
Effect of mercurials and HDACis on developmental genes (D-genes) in the UKK and UKN1 systems. Differentiating cells were treated by mercurials and HDACis as indicated in Fig. 1a and were used for transcriptome analysis. Genes affected by the differentiation process (D-genes) were identified, as shown in Fig. 2, as well as toxicant-affected genes (T-genes), as shown in Fig. 4. **a** The overlap of up-regulated mercurial T-genes with up-(*red*) and down-(*blue*) regulated D-genes as well as the overlap of down-regulated mercurial T-genes with up- and down-regulated D-genes was calculated for each system. The data are expressed as the fraction of D-genes affected by toxicants. **b** The same procedure was performed for the HDACis. Blue bars represent D-genes down-regulated and *red bars* indicate D-genes up-regulated during normal differentiation. The *numbers* on top of the *bars* indicate the absolute number of PSs affected (colour figure online)

To analyse the relationship of chemically influenced PSs (further referred as genes) and developmental PSs (further referred as genes), we next focused on the pool of genes deregulated by at least one of the tested chemicals, as indicated by the red frame in Fig. 4c (Suppl. Table 5). Overlap analysis between developmental and chemically deregulated genes revealed a strongly compound-dependent and test system-dependent pattern (Fig. 6a, b). In the UKK system, genes deregulated by mercurials (by at least one of the six tested compounds) showed only very little overlap with developmental genes (Fig. 6a). In contrast, a very high degree of overlap of more than 60 % was observed in the UKN1 test system. This shows that the developmental genes in the UKN1 system are more susceptible to mercurials (Fig. 6a) and HDACis (Fig. 6b) compared with those of the UKK system. In contrast with mercurials, HDACis deregulated developmental genes in both the UKK and UKN1 systems, although the degree of overlap between compound-associated and developmental genes was also higher in the UKN1 system (Fig. 6b). Furthermore, a characteristic feature is that most of the chemically influenced genes in the overlap antagonize the spontaneous developments in differentiating hESCs; genes that are induced during development are suppressed by exposure to chemicals, while decreased gene expression is induced. The opposite constellation, the chemical induction of increased developmental gene expression or the further suppression of already spontaneously decreasing gene expression, was comparatively rare (Fig. 6). The analysis shown in Fig. 6 is based on developmental genes that are at least fivefold up- or down-regulated. In a similar analysis, we differentiated developmental genes deregulated by two-, five-, and tenfold (absolute values, Suppl. Fig. S4). This analysis confirmed the concept that developmental toxicants usually antagonize the spontaneous up- or down-regulation of developmental genes, similar to that shown in Fig. 6.

In conclusion, mercurials or HDACis usually antagonize the spontaneous development of gene expression in differentiating hESCs. This characteristic feature was also shown by an alternative approach, in which high and low gene expression was differentiated using a log2 value of 6 as a threshold (Suppl. Fig. S5; Suppl. Table 6). An overlap of chemically up-regulated genes mostly occurs for genes that are high in the undifferentiated hESCs and decrease during the differentiation period (Suppl. Fig. S5A, B), while chemically induced down-regulated genes overlap with developmental genes that are lowly expressed in stem cells and have increased expression towards the end of the in vitro differentiation period. Reciprocal scenarios were only rarely observed (Suppl. Fig. S5).

### Developmental potency (*D*_p_) and developmental index (*D*_i_) for the characterization of compromised stem cell development

**Fig. 7:**
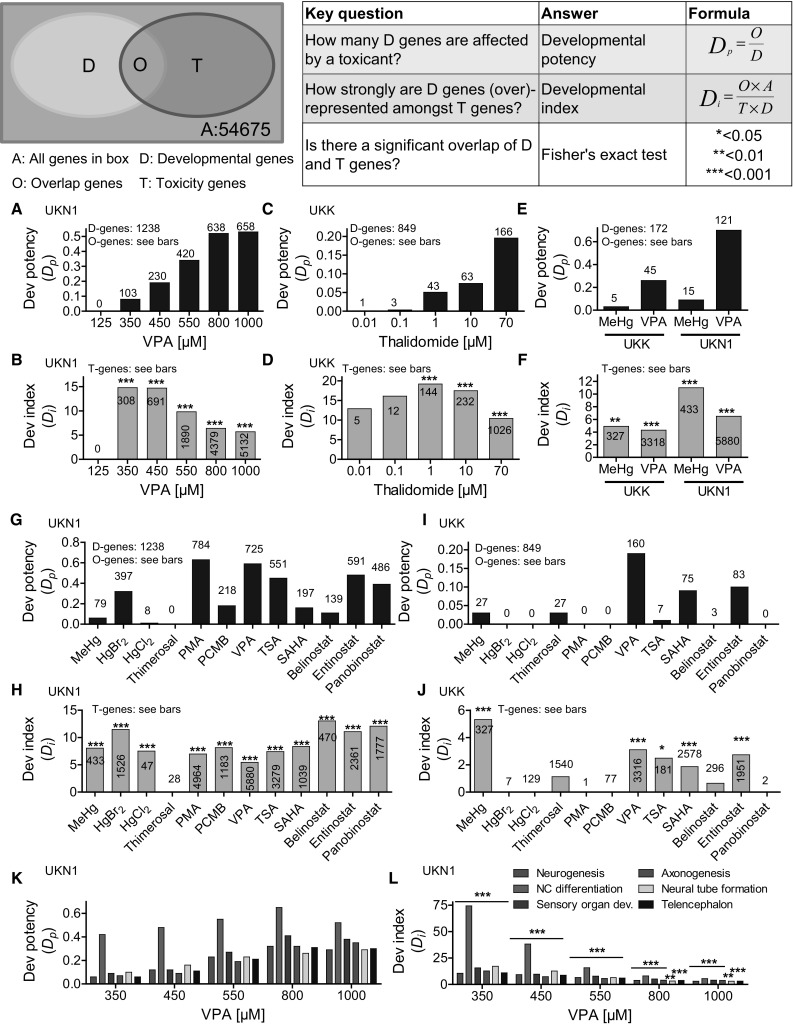
Design of transcriptome-based developmental potency (*D*_p_) and developmental index (*D*_i_) to characterize toxicant-disturbed stem cell development. The scheme on top exemplifies the number of developmental PSs (*D*; *yellow circle*) that are significantly deregulated during stem cell differentiation and the number of toxicity PSs (*T*; *blue circle*) that are significantly deregulated by a compound. In total, 54,675 probes are measured on the microarray chip (*grey box*). D-genes (or respective PSs) that are significantly deregulated by a toxicant are classified as overlap PSs/genes (*O*; *green*). The parameters from the scheme are used to calculate developmental potency (*D*_p_) and developmental index (*D*_i_); the corresponding formula and key questions are given. Fisher’s exact test was used to determine whether there was a significant overlap of D- and T-genes. The questions were applied to various toxicity testing experiments. **a**–**d** The list of PSs influenced by various concentrations of valproic acid (VPA) in the UKN1 test system and thalidomide in the UKK test system was retrieved from the literature (Meganathan et al. 2012; Waldmann et al. 2014), and *D*_p_ and *D*_i_ were calculated and plotted. **e**, **f** *D*_p_ and *D*_i_ were calculated for methylmercury (MeHg) and VPA in the UKK and UKN1 test systems. As a reference basis for the D-genes, the common D-genes of the UKK and UKN1 systems (as defined in Fig. 2a) were used instead of the D-genes specific for each system. **g**, **h** *D*_*p*_ and *D*_i_ were calculated for six mercurials and six HDACis in the UKN1 and (**i**, **j**) the UKK test systems using system-specific D- and T-genes. The total number of D-genes (*green*) and T-genes (*purple*) are given on top of the *bars*. **l**, **m** *D*_p_ and *D*_i_ were calculated for specific differentiation processes for VPA in the UKN1 test system, as indicated. Detailed lists of all compound-deregulated PSs, developmental PSs and PSs belonging to the specific differentiation processes in the UKK and UKN1 test systems are provided in supplemental materials. *p < 0.05; **p < 0.01; ***p < 0.001 for D/T overlap according to Fisher’s exact test, with T-genes as indicated in the *bar graph* and D-genes for the respective test systems (colour figure online)

The analyses of the previous paragraph showed that chemicals deregulate developmental genes in a complex manner. Investigations such as those shown in Figs. 1, 2, 3, 4, 5, 6 may be too complex for routine toxicological studies. To reduce complexity, we propose two indices that are relatively easy to obtain and yet correctly describe how chemicals interfere with the transcriptome of differentiating stem cells. The most important index is ‘developmental potency’ (*D*_p_), which represents the fraction of all developmental genes that are altered by a test compound at a given concentration. For the example of VPA in the UKN1 system (Fig. 7a; Suppl. Table 7), *D*_p_ increases in a concentration-dependent manner until the expression of more than 50 % of all developmental genes is compromised at the highest tested concentrations of 800 and 1000 µM. Although the altered expression of developmental genes as indicated by increased *D*_p_ is already sufficient to objectify a hazard, we nevertheless recommend to additionally consider a second index, the ‘developmental index’ (*D*_i_). *D*_*i*_, calculated as shown in Fig. 7, yields information about the ratio by which developmental genes are overrepresented among the genes deregulated by a chemical. If *D*_i_ is 1.0, the fraction of developmental genes of all genes deregulated by the test compound corresponds to what can be randomly expected. If *D*_i_ increases to values higher than 1.0, the chemical preferentially influences developmental genes. The formula for calculating *D*_i_ normalizes it to the total number of developmental genes (Fig. 7). Therefore, data from distinct cell systems such as UKK and UKN1 with different numbers of developmental genes can be directly compared. The example of VPA in the UKN1 system shows that already, the lowest concentration of 350 µM, which leads to significantly deregulated genes, shows a *D*_i_ increased by approximately 15-fold (Fig. 7b). Interestingly, *D*_i_ decreased with increasing concentrations of VPA; this decrease occurred because concentrations of 550 µM VPA and higher caused the expression of cell death-associated genes (Waldmann et al. 2014) that do not overlap with developmental genes. Testing very high, nearly cytotoxic concentrations can therefore lead to decreased *D*_i_; however, it should be considered that even at the highest tested cytotoxic concentration of 1000 µM VPA, *D*_i_ was still increased by more than fourfold. An interesting scenario of *D*_p_ and *D*_i_ was obtained when the teratogenic thalidomide was tested in the UKK system (Fig. 7c, d; Suppl. Table 8). The lowest tested thalidomide concentrations of 0.01 and 0.1 µM showed only a slightly increased *D*_p_ because only a few genes were deregulated at these concentrations. However, *D*_i_ was already high because a high fraction of the small number of deregulated PSs was developmental genes. Therefore, 0.01 and 0.1 µM thalidomide should be already considered as potentially hazardous developmentally and teratogenic. Similarly, the benchmark concentrations for MeHg and VPA would be interpreted as hazardous following the aforementioned rules of interpretation (Fig. 7e, f). The concept of the two developmental indices also allows a clear interpretation of the 12 mercurials and HDACis (Fig. 7g–j). It is also clear that the UKN1 system shows a higher susceptibility than does the UKK system for both *D*_p_ and *D*_i_. As shown in Fig. 7g–j, some compounds resulted in negative test data because no genes were significantly deregulated at the tested concentrations. If a test compound does not induce any gene expression alterations over a 6- or 14-day incubation period, considering these test conditions as non-hazardous may be justified.

The concept of these developmental indices can be further refined by differentiating biological categories of developmental genes. The example of VPA in the UKN1 system illustrates that for both *D*_p_ and *D*_i_, neural crest cell differentiation was compromised to a higher degree than were other categories, such as neurogenesis in general, sensory organ development, axonogenesis and telencephalon development (Fig. 7k, l; Suppl. Table 7 and 9). With increasing concentrations, the predominance of neural crest differentiation decreased, probably because genes of other biological categories also became increasingly deregulated. In contrast with the UKN1 system, the UKK test system shows increased *D*_p_ and *D*_i_ for a much broader range of categories, including limb, heart, skin and liver development after incubation with thalidomide (thalidomide and all further exposure conditions: Suppl. Fig. S6; Suppl. Table 8 and 10).

### Identification of diagnostic genes

**Fig. 8:**
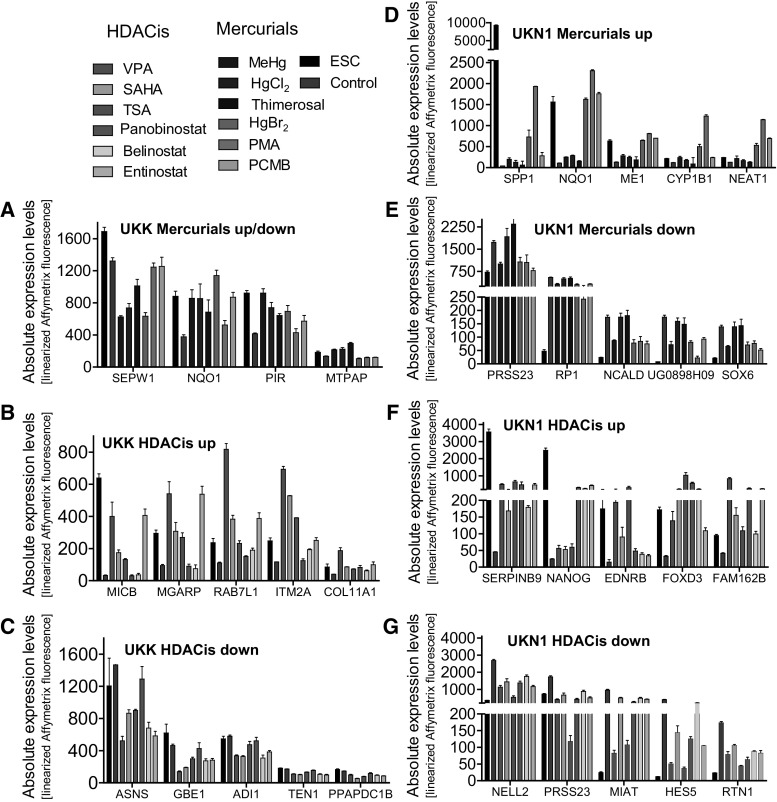
Identification of mercurial- and HDACi-induced diagnostic genes in the UKK and UKN1 test systems. For the identification of the top diagnostic genes, the following selection criteria were used, as detailed in the ‘Materials and methods’ section: (1) regulation by several compounds within a toxicant class; (2) baseline expression values clearly higher than the Affymetrix noise range; (3) genes showing disease association by the online tool ‘DAVID’; and (4) literature evidence for disease association in animal models and humans. **a** Four diagnostic genes were affected by mercurials in the UKK test system. Five diagnostic genes were up-(**b**) and down-(**c**) regulated by HDACis in the UKK test system. Five diagnostic genes were up-(**d**) and down-(**e**) regulated by mercurials in the UKN1 test system. Five diagnostic genes were up-(**f**) and down-(**g**) regulated by HDACis in the UKN1 test system. The* colours* indicate the toxicant used; *black bars* give expression levels in undifferentiated H9 cells; *green bars* give expression levels in untreated, differentiated control cells. The data are shown as the mean ± SD; *n* = 4 (colour figure online)

For the identification of developmental toxicants, the availability of diagnostic genes is of high relevance. The top diagnostic genes shown in Fig. 8 were selected from the consensual PSs (Fig. 4c; green and orange fields) based on the additional criteria as mentioned in the materials and method section. The baseline expression cutoff value has been set to be >6 (log2 scale) on day 0 or day of differentiation based on the frequency distribution curves obtained with base line expression values (log2 scale) for all PSs on microarray (54675) of non-treated control samples on day 0 and day 6 or day 14 (Suppl. Fig. S5C & D). The overlap analysis of mercurial and HDACis deregulated PSs in UKN1 test system with the baseline expressed values (log2 scale) revealed that major overlap obtained with compound-deregulated PSs is with the PSs whose baseline expressed values were >6 (log 2 scale) (Suppl. Fig. S5A & B).

### Identification of gene–disease association

For identification of gene–disease association, the consensus genes (Suppl. Table 4) were uploaded into the online tool ‘DAVID’, and genes showing disease associations were filtered out. The literature was searched for whether loss-of-function or gain-of-function mutations are known for these genes and whether an association with disease had been documented in in vivo models as well as in humans. The results are presented in Tables 2 and 3. Further information was also obtained for the genes reported to be de-regulated by the mercurials or HDACis in in vitro studies and their presence in consensus genes (mercurials, Table 2; HDACis, Table 3).

**Table 2:** Biological or disease relevance of mercurial consensus genes

Gene	Mercurial	Known literature data	References	Relationship with known toxicity
*MTPAP* (Mitochondrial poly(A) polymerase)	Up (UKK)	Mutated in human autosomal-recessive neurodegeneration, *spastic ataxia*	Crosby et al. (2010)	*In utero exposure of MeHg in humans*: resulted in *ataxia, spastic paraparesis*, learning difficulties, speech and walking delay; histopathology—*ectopic cell masses in the cortex*, disorganized layers in the brain; Long-lasting effects on vision in humans, rat off springs (Burbacher et al. 1990)
*SOX6* (SRY (sex determining region Y)-box 6)	Down (UKN1)	In mouse required for normal positioning and maturation of cortical interneuron subtypes	Batista-Brito et al. (2009)	
*THBS1* (Thrombospondin 1)	Up (UKN1)	It promotes neurites outgrowth in culture, affects migration of neuronal precursor cells in vivo in developing brain.	Blake et al. (2008), Lu and Kipnis (2010)	
*CYP1B1* (Cytochrome P450, family 1, subfamily B, polypeptide 1)	Up (UKN1)	Mutation in *congenital glaucoma* in human; role in murine embryonic axis development, neural tube patterning in chick embryo, overexpression potentiates retinal ganglion cell survival	Badeeb et al. (2014), Chambers et al. (2007), Stoilov et al. (2004), Wang et al. (2007)	
*NQO1* (NAD(P)H dehydrogenase, Quinone 1)	Up (UKK, UKN1)	Up-regulated/polymorphism in *Alzheimer’s disease* in humans. Induced by MeHg in primary rat microglial cells	Luo et al. (2016), Ni et al. (2010), Raina et al. (1999)	MeHg toxicity associated with *neurodegenerative diseases* in in vivo models and in humans. Mutter et al. (2010), Pendergrass et al. (1997)
*SPP1* (Secreted phosphoprotein 1/osteopontin)	Up (UKN1)	Up-regulated in *autistic children*, involved in autoimmune neuroinflammatory disease & multiple sclerosis	Al-ayadhi and Mostafa (2011), Chabas et al. (2001)	
*UCHL1* (Gamma-aminobutyric acid (GABA) a receptor, beta 3)	Up (UKN1)	Mutated in *Parkinsons and Alzheimers* in humans	Wobst et al. (2012), Yasuda et al. (2009)	
*GABRB3* (Ubiquitin carboxyl-terminal esterase L1)	Up (UKN1)	mutated in neurodevelopmental disorder *austism spectrum* conditions in humans	Warrier et al. (2013)	
*NPY2R* (Neuropeptide Y receptor Y2)	Down (UKN1)	It has neuroprotective effect in vitro and in vivo animal models of *Parkinson’s disease*	Decressac et al. (2012)	
*FTL* (Ferritin, light polypeptide)	Up (UKN1)	Mutated in neurodegenerative disease—*hereditary ferrinopathy* (tremors, cerebellar signs, cognitive defects etc.)	Kubota et al. (2009), Vidal et al. (2004).	
*SEPW1* (Selenoprotein W1)	Down (UKK)	It protects neurons from oxidative stress during rat neuronal development; role in brain development, expressed in hippocampus, cortex, cerebellum and olfactory bulb	Chung et al. (2009), Kim et al. (2005), Pitts et al. (2014)	*Glutathione enzyme* and *selenoproteins* are evaluated as mercury *biomarkers* in humans. Goodrich et al. (2013)
*GCLM* (Glutamate–cysteine ligase modifier subunit)	Up (UKN1)	MeHg binds to thiol group regulatory protein (Keap 1) of Nrf and its activation induces up-regulation of GCLM in human neuroblastoma cells	Toyama et al. (2007)	
*SLC7A5* (Solute carrier family 7 (amino acid transporter light chain, L system))	Up (UKN1)	linked with *autism*, Encodes transporter LAT1 which increases transport of l-Cysteine conjugated MeHg in cells	Anderson et al. (2009), Yin et al. (2008)	
*MSX2* (Msh homeobox 2)	Up (UKN1)	Gain in function mutation-*craniosynostosis* (premature fusion of calvarial bones of skull in human)	Ma et al. (1996), Wuyts et al. (2000)	
*COL1A2* (Collagen, type I, alpha 2)	Up (UKN1)	Mutated in *osteogenesis imperfecta type I*–*IV* in humans	Pollitt et al. (2006), Ward et al. (2001)

**Table 3:** Biological or disease relevance of HDACis consensus genes

Gene	HDACi	Known literature data	References	Relationship with known toxicity
*ASNS* (Asparagine synthetase (glutamine-hydrolysing))	Down (UKK)	Mutations found in *congenital microcephaly* human	Ruzzo et al. (2013)	VPA-induced *congenital microcephaly* in humans with craniofacial abnormalities: short nose, philtrum of lip, micrognathia, urogenital anomalies Ardinger et al. (1988) *Facial dysmorphism*, craniosynostosis, neural tube defects, Chandane and Shah (2014) *Atrial septum defects*, ventricular septum defects, tetralogy of fallot observed in foetal valproate syndrome. Jentink et al. (2010) *Genitourinary tract defects*, *autism-*related cerebellar anomalies in humans and rats. Ingram et al. (2000) VPA-induced neurite outgrowth linked with *autism* spectrum disorder (Chomiak et al. (2013)) Multiple *ocular associations* **—**strabismus, myopia, nystagmus, epicanthic folds, infraorbital creases and dry eye and nasolacrimal duct obstruction etc. (Hornby and Welham 2003)
*COL11A1* (Collagen, type XI, alpha 1)	Up (UKK)	Mutation found in *Stickler syndrome* patients	Annunen et al. (1999), Richards et al. (1996)	
*GBE1* (Glucan (1,4-alpha-), branching enzyme 1)	Down (UKK)	Mutation in human patients results in *adult polyglucosan body disease* and glycogen storage disease IV in equines	Sampaolo et al. (2015), Ward et al. (2004)	
*EDNRB* (Endothelin receptor type B)	Up (UKN1)	Mutation associated with *Hirschsprung disease type II and ABCD syndrome*, restricted period expression in neural crest development in mouse	McCallion and Chakravarti (2001), Shin et al. (1999)	
*MIAT* (Myocardial infarction associated transcript)	Down (UKN1)	SNPs found in *myocardial infarction and schizophrenia* in humans	Ishii et al. (2006), Rao et al. (2015)	
*PRSS23* (Protease, serine, 23)	Down (UKN1)	knock down result in *atrioventricular valve defect* in zebrafish	Chen et al. (2013)	
*RTN1* (Reticulon 1)	Down (UKN1)	RTN1 helps in vesicular transport of Spastin and disturbance of this process probable cause of *Hereditary Spastic Paraplegias*	Mannan et al. (2006)	
*FOXD3* (Forkhead box D3)	Up (UKN1)	variants found in *Aniridia, Peters anomaly, anophthalmia* in humans	Kloss et al. (2012)	
*GABRB2* (GABA-A receptor, beta 2)	Up (UKK)	Mutation results in *congenital intellectual disability* in human patients	Srivastava et al. (2014)	
*ARNT2* (Aryl-hydrocarbon receptor nuclear translocator 2)	Down (UKN1)	Loss-of-function mutation in humans results in *Webb*–*Dattani syndrome*	Webb et al. (2013)	
*DNER* (Delta/Notch-like EGF repeat containing)	Up (UKN1)	Overexpression inhibits proliferation of neural progenitors in zebrafish; Mutation in mouse impairs cerebellar functions	Hsieh et al. (2013), Tohgo et al. (2006)	
*CASP8* (Caspase 8, apoptosis-related cysteine peptidase)	Up (UKN1)	Conditional knockout attenuates *neural tube defects* in mouse. Involved in *Alzheimer’s, Parkinson’s and Huntington’s Disease*	Ahmad et al. (2014), Yang et al. (2013)	

## Discussion

Recently, hESC-based in vitro systems that recapitulate specific phases of human development have become available (Balmer et al. 2014; Krug et al. 2013; Zimmer et al. 2014). In UKN1 and UKK test systems genome-wide expression data of 12 compounds (six HDACi and six mercurial) was obtained for benchmark concentrations (BMC10) resulting in viability reduced by a maximum of 10 %. The test compound VPA was additionally tested over a wide range of concentrations, from non-toxic up to severely cytotoxic concentrations. Additionally, the teratogen thalidomide was studied over a wide concentration range using the UKK test system. Based on this genome-wide data set, we studied the basic principles of how chemicals interfere with gene expression in differentiating stem cells. One key feature is that chemicals antagonize the spontaneous developments of gene expression in differentiating stem cells. Genes up-regulated during differentiation were suppressed, while down-regulated genes were induced when exposed to the test compounds. A second key feature is that compounds differ widely in the fraction of developmental genes whose expression they compromise. For example, VPA influences a higher fraction of developmental genes in both test systems than does HgCl_2_, although both compounds were tested at benchmark concentrations (BMC10). A third feature is the difference in susceptibility between both test systems. For example, a higher fraction of developmental genes is compromised by MeHg and VPA in the UKN1 system than in the UKK test system. Because the UKN1 system recapitulates the formation of neuronal precursor cells and the UKK system recapitulates the development of the three germ layers and their derivatives, it can be expected that the latter process is less susceptible to the analysed compounds.

Considering the above-mentioned features, we recommend two indices to quantify the developmental toxicity potential of different compounds, the developmental potency index (*D*_p_) and the developmental index (*D*_i_). *D*_p_ gives the intersection of the genes between the deregulated genes (up- or down-regulated) by a test compound and the genes deregulated at day 6 (UKN1) or day 14 (UKK) of differentiated versus undifferentiated hESCs in the absence of a test compound. A high *D*_p_ can be interpreted as a high hazard of toxicity. It should be considered that the total number of deregulated genes is associated with *D*_p_. If a compound deregulates only a small number of genes, e.g. *n* = 20, *D*_p_ will inevitably be small. However, *D*_p_ per se might not discriminate developmental toxicity effects occurring on the expression of genes required for the differentiation of hESCs to neural progenitor cells (UKN1) or for the formation of germ layer cells and their derivatives (UKK) from general toxicity effects not related to differentiation processes (e.g. toxicity effects occur also in differentiated cells). To discriminate developmental toxicity effects, we recommend additionally considering the developmental index *D*_i_. This index provides information about the ratio by which developmental genes are overrepresented among ‘toxicity genes’, which represents the number of genes deregulated by the test compound. If *D*_i_ is high, developmental genes are overrepresented among ‘toxicity genes’. A low *D*_i_ shows that developmental genes are underrepresented because the compound preferentially influences biological processes not related to development. The formula of *D*_i_ has been designed to consider the number of developmental genes. Therefore, test systems with different numbers of developmental genes can be directly compared. The relevance of *D*_i_ can be illustrated by the example of the teratogenic compound thalidomide (Fig. 7c, d). The very low concentration of 0.1 µM thalidomide up-regulates only 12 genes, resulting in a very low *D*_p_, which does not indicate a severe hazard effect. However, *D*_i_ for this test condition is approximately 16, meaning that thalidomide specifically compromises the expression of developmental genes with a ratio 16-fold higher than can be randomly expected. Therefore, 0.1 µM thalidomide may be considered as developmentally hazardous, even though the expression of only a few genes is compromised. An opposing trend to thalidomide can be illustrated by thimerosal. In the UKK system, thimerosal deregulated 1540 PSs, which similar to 0.1 µM thalidomide, resulted in a low *D*_p_. However, a *D*_i_ of 1.16 for thimerosal indicates that developmental genes are not overrepresented among the PSs deregulated by this compound (Fig. 7i, j). In conclusion, using these systems with *D*_p_ and *D*_i_ gives a rapid overview of developmental toxicity. If one of the two indices is high, the test condition should be interpreted as hazardous. Moreover, hazardous compounds can be classified as developmentally and less developmentally hazardous compounds. In conclusion, based on this approach with hESCs and transcriptomic technologies, we have established two very sensitive test systems allowing the discrimination of developmental (teratogenic) toxicity from non-developmental, general toxicity hazards: the ‘STOP-Tox_ukn_’ and the ‘STOP-Tox_ukk_’ tests (Stem cell-based Teratogenic Omics Prediction; UKN: University of Konstanz; UKK: Universitätsklinikum Köln).

Comparing the results of the two compound classes, it should be considered that the selected HDACis and mercurials showed different degrees of homogeneity. HDACis that have been discussed previously (Rempel et al. 2015; Yang et al. 2013) represent a relatively homogenous compound group. This is illustrated by a consensus of 90 up- and 18 down-regulated genes that are influenced by all six HDACis in the UKN1 system. In contrast, mercurials showed a lower degree of overlap of deregulated genes. No single gene that was influenced by all six mercurials could be identified. Nevertheless, there is a set of consensus genes influenced by five of six mercurials, which are appropriate to use for the identification of developmental toxicity in the two test systems.

A further goal of this study was to identify individual genes that are particularly suitable to use for the prediction of developmental toxicity using the test systems STOP-Tox_ukk_ and STOP-Tox_ukn_, further referred to as ‘diagnostic genes’ for severe developmental defects in humans. Prenatal exposure to teratogens results in congenital disorders in humans, which are known to be related with mutations in various genes (Webber et al. 2015). The disease association of the consensus genes de-regulated by mercurials or HDACis further helps to understand mechanistic aspects. The selection criteria were that diagnostic genes should be influenced by as many compounds of the same class (i.e. HDACis and mercurials) as possible; they should show the highest fold changes compared with the controls; they should antagonize the expression of developmental genes; and their biological relevance should be clear, based on animal knockout or overexpression models or on human disease data. Based on these criteria, several top diagnostic genes were identified.

The significance of this association can be related to the fact that mercurials can perturb neurodegenerative genes by epigenetic events, resulting in altered gene expression and late onset of neurodegenerative diseases. Mercurial consensus genes that are related to genetic disease associations are the following: *MTPAP, SEPW1* (UKK); *NQO1* (UKK/UKN1)*; SPP1, CYP1B1, GABRB3, UCHL1, SLC7A5, COL1A2 and FTL* (UKN1). The only up-regulated gene found in the UKK test system was *MTPAP*. Interestingly, *a* mutated *MTPAP* has been reported in human autosomal-recessive spastic ataxia (Crosby et al. 2010). Moreover, the polymorphisms or overexpression of the detoxification enzyme NQO1 has been reported in Alzheimer’s patients (Raina et al. 1999) and was captured by mercurials in both systems. The up-regulation of *SPP1* has been observed in autistic children (Al-ayadhi and Mostafa 2011). Mutations of *GABRB3* have been identified and a role of the *SLC7A5* gene has also been described in autistic patients (Anderson et al. 2009; Warrier et al. 2013). Mutations in *UCHL1* have been identified in Parkinson’s patients (Liu et al. 2015), whereas a gain-of-function mutation in the corresponding mouse gene resulted in Parkinson’s disease symptoms (Yasuda et al. 2009). A mutation in *FTL* resulted in neurodegenerative hereditary ferrinopathy, characterized by tremor and cognitive defects (Kubota et al. 2009; Vidal et al. 2004). The *NPY2R* gene has been shown to serve in a neuroprotective role in animal models of Parkinson’s disease (Decressac et al. 2012). Notably, *NPY2R* was found to be down-regulated by mercurials.

Autopsies of children exposed to MeHg in utero showed degeneration and atrophy of cortical structures, ectopic cell masses in the cortex, astrocytes present in white matter and cell loss prominent in the cerebellum and cerebrum (Burbacher et al. 1990). The biological functions of two mercurial consensus genes *SOX6* and *THBS1* are in agreement with these pathologies. *SOX6* was found to be down-regulated in our study, and a *SOX6* mouse knockout showed that expression is required for normal positioning and maturation of cortical interneuron subtypes (Batista-Brito et al. 2009). *THBS1* was up-regulated by the mercurials and has been shown to promote neurite outgrowth as well as post-natal migration of neuronal precursor cells in mice (Liu et al. 2015). Additional diagnostic mercurial consensus genes are *SEPW1* and *GCLM*. *SEPW1* has been reported to be a neuroprotectant, and it is targeted by MeHg in human neuronal cells (Kim et al. 2005). It has also been reported that MeHg up-regulates *GCLM* in human neuroblastoma cells (Toyama et al. 2007). Recently, a good correlation between polymorphisms of *SEPW1* and *GCLM* and the potential of eliminating mercurials in urine and hair has been reported in humans (Goodrich et al. 2011, 2013).

In animal models, inhibition of HDAC resulted in altered gene expression during embryonic development that was accompanied by congenital malformations (Menegola et al. 2005). The data from the literature suggest that VPA-induced malformations in humans are congenital microcephaly, atrial and ventricular septal defects, craniofacial abnormalities, neural tube defect spina bifida, urogenital abnormalities and ear abnormalities (Ardinger et al. 1988). HDACis regulated consensus genes that are linked to these malformations include *ASNS, COL11A1, GABRB2,* and *GBE1* in the UKK system and *RTN1, SMOC1, RAX, ARNT2, CASP8, MIAT, BMP5* and *EDNRB* in the UKN1 test system. *ASNS* was found down-regulated by the HDACis. In this context, it has been reported that recessive loss-of-function mutations of *ASNS* in patients caused congenital microcephaly, intellectual disability and progressive cerebral atrophy. It has also been demonstrated that asparagine depletion due to *ASNS* deficiency results in neurological impairment in knockout mice (Ruzzo et al. 2013). Mutations in the *COL11A1* gene have been found in patients with Marshall syndrome and Stickler syndrome, which share similar phenotypes, such as craniofacial abnormalities, a flat nasal bridge, midface hypoplasia, a short nose, palate defects and hearing loss (Annunen et al. 1999). Mutations in *GBE1* have been detected in patients of adult polyglucosan body disease with symptoms such as progressive gait difficulty, urinary incontinence, and reduced reflexes in lower limbs (Sampaolo et al. 2015). In the present study, *COL11A1* and *GBE1* were found to be down-regulated by the incubation of the hESCs with the HDACis. Mutations in *EDNRB* have been identified in patients suffering from Hirschsprung’s disease, which is characterized by the absence of neural crest-derived intramural ganglia along the colon (McCallion and Chakravarti 2001). *MIAT* was found to be down-regulated in the present study. Although this gene is not translated to protein, it transcribes non-coding functional RNA. Recently, SNPs of *MIAT* have been identified in human patients with myocardial infarction and schizophrenia (Ishii et al. 2006; Rao et al. 2015).

*PRSS23*, which was found to be down-regulated in our study, encodes for a novel serine protease that is expressed during the development of the cardiovascular system, and it is involved in valvulogenesis of zebrafish. *PRSS23* plays a significant role in the endothelial to mesenchymal transition during valvulogenesis, and the knockdown of the gene results in atrioventricular defects (Chen et al. 2013). *FOXD3* was found to be up-regulated, and it is linked with congenital eye deformities such as aniridia, Peter’s anomaly and anophthalmia in humans (Kloss et al. 2012). *RTN1* plays a role in the vesicular transport of Spastin, which is involved in microtubule dynamics. The disruption of Spastin-mediated vesicular transport has been discussed as a cause of hereditary spastic paraplegias, with symptoms such as the progressive stiffness of lower limbs due to nerve dysfunction, cognitive impairments and deafness (Mannan et al. 2006). Other diagnostic consensus genes with disease relevance are *GABRB2, ARNT2, DNER* and *CASP8*. The overexpression of *DNER* inhibits the proliferation of neural progenitor cells and induces glial cell differentiation in zebrafish (Hsieh et al. 2013). A loss-of-function mutation of *DNER* in mice impairs cerebellar functions (Tohgo et al. 2006). *ARNT2* was found to be down-regulated in the present study. A loss-of-function mutation of *ARNT2* in humans results in Webb–Dattani syndrome, with characteristic features such as microcephaly, visual impairment and urinary abnormalities (Webb et al. 2013). Caspase 8 was up-regulated by the HDACis and has been described to regulate neuronal apoptosis involved in neurodegenerative diseases such as Alzheimer’s, Parkinson’s and Huntington’s disease in humans (Ahmad et al. 2014). In conclusion, the involvement of the diagnostic genes deregulated by mercurials and HDACis by both test systems in developmental toxicity in humans and experimental animals has been well established.

A limitation of the present study is the relatively small number of test compounds. A next important step will be to include compounds with very low cytotoxicity (‘negative controls’) as well as cytotoxic compounds that are known to cause adverse effects other than developmental toxicity. The extrapolation from a hazardous test condition in vitro, e.g. defined by a high *D*_p_ or *D*_i_, to adverse developmental effects in vivo remains difficult. One challenge is to simulate the concentrations of a test compound at the target cell of toxicity in vivo. Although physiologically based pharmacokinetic (PBPK) modelling addresses this aspect, its predictions are still associated with a relatively high degree of uncertainty. A further limitation that has to be taken into account is that in vitro systems do not necessarily recapitulate all in vivo relevant mechanisms. Despite these limitations, the concept proposed here with developmental potency and ratio indices quantitatively provides information regarding the propensity of test compounds to interfere with the complex transcriptional process required for normal human development. In conclusion, this study offers a concept for the identification of potential developmental toxicity hazards by establishing *D*_p_ and *D*_i_, which predict the propensity of test compounds to interfere with transcriptional processes during human development.

## Electronic supplementary material

Below is the link to the electronic supplementary material.

Supplementary material 1 (PPTX 2756 kb)

Supplementary material 2 (XLSX 5359 kb)
