# Supplementary material for: Definition of transcriptome-based indices for quantitative characterization of chemically disturbed stem cell development: introduction of the STOP-Toxukn and STOP-Toxukk tests
Source: Arch Toxicol. 2016 May 17;91(2):839–64. doi: 10.1007/s00204-016-1741-8 (PMC5306084; doi:10.1007/s00204-016-1741-8)
Supplement: Supplementary file 1 — Supplementary material 1 (PPTX 2756 kb) [file 204_2016_1741_MOESM1_ESM.pptx]

## Slide 1
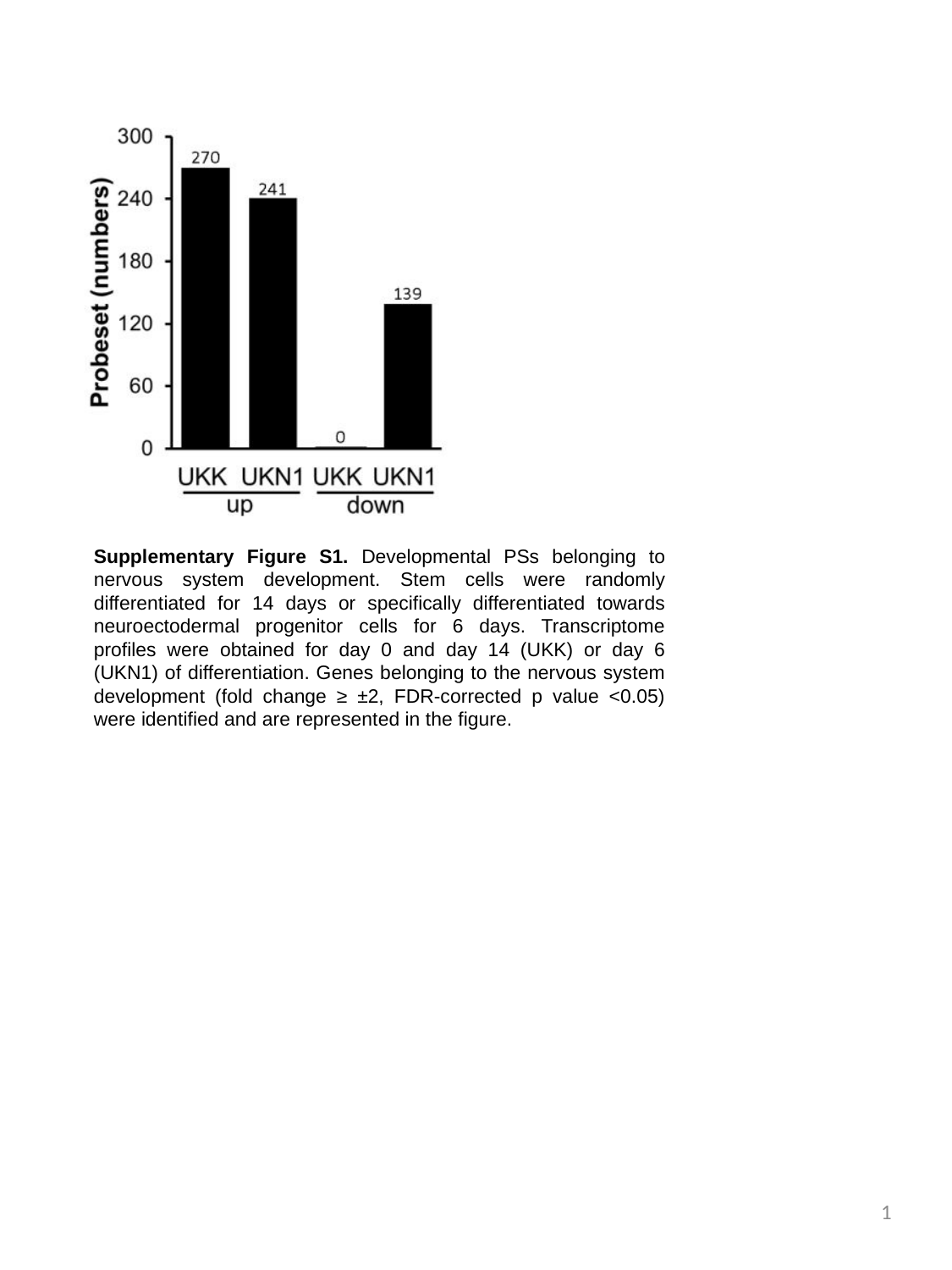

Supplementary Figure S1. Developmental PSs belonging to nervous system development. Stem cells were randomly differentiated for 14 days or specifically differentiated towards neuroectodermal progenitor cells for 6 days. Transcriptome profiles were obtained for day 0 and day 14 (UKK) or day 6 (UKN1) of differentiation. Genes belonging to the nervous system development (fold change ≥ ±2, FDR-corrected p value <0.05) were identified and are represented in the figure.
1

## Slide 2
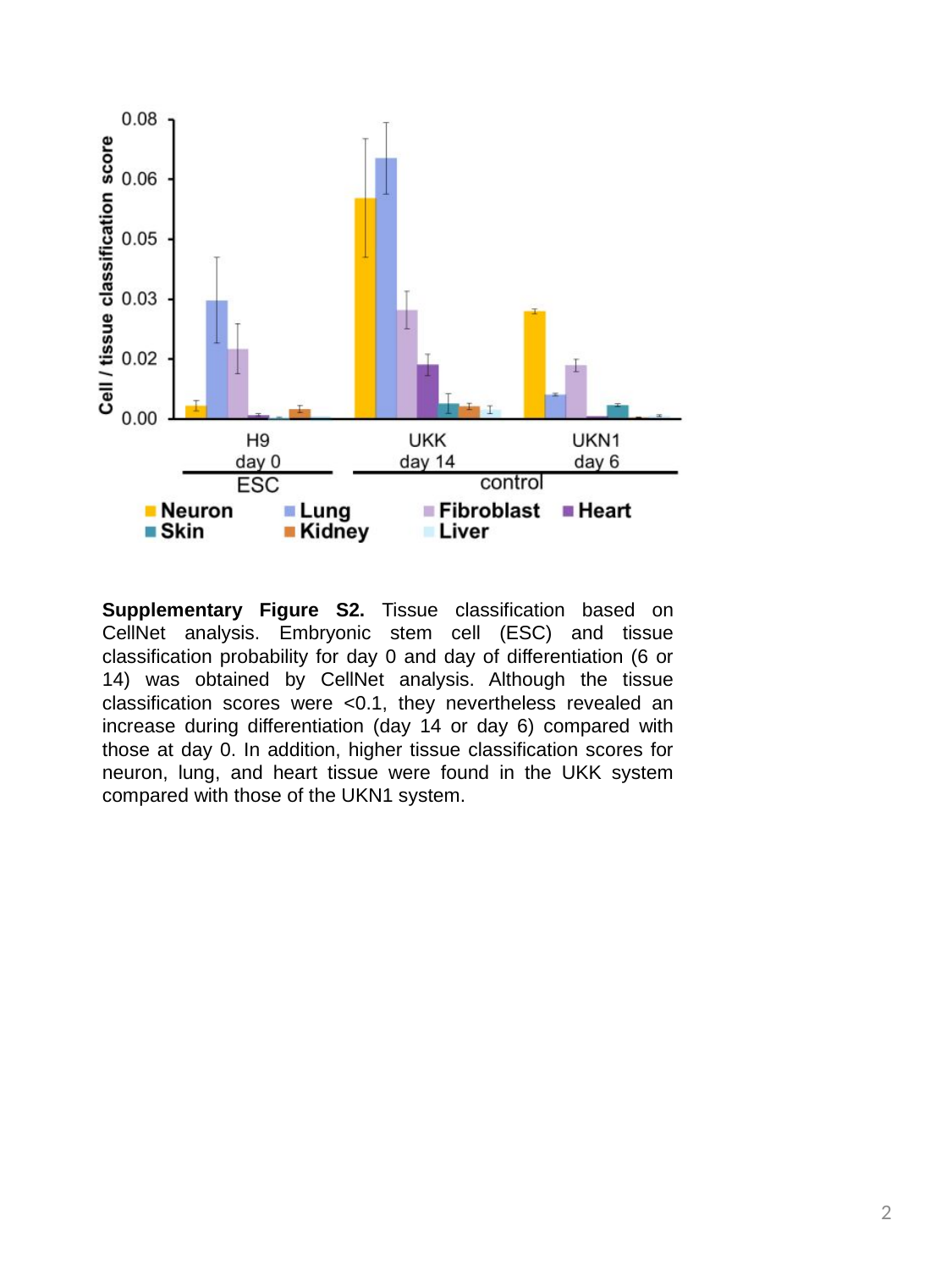

Supplementary Figure S2. Tissue classification based on CellNet analysis. Embryonic stem cell (ESC) and tissue classification probability for day 0 and day of differentiation (6 or 14) was obtained by CellNet analysis. Although the tissue classification scores were <0.1, they nevertheless revealed an increase during differentiation (day 14 or day 6) compared with those at day 0. In addition, higher tissue classification scores for neuron, lung, and heart tissue were found in the UKK system compared with those of the UKN1 system.
2

## Slide 3
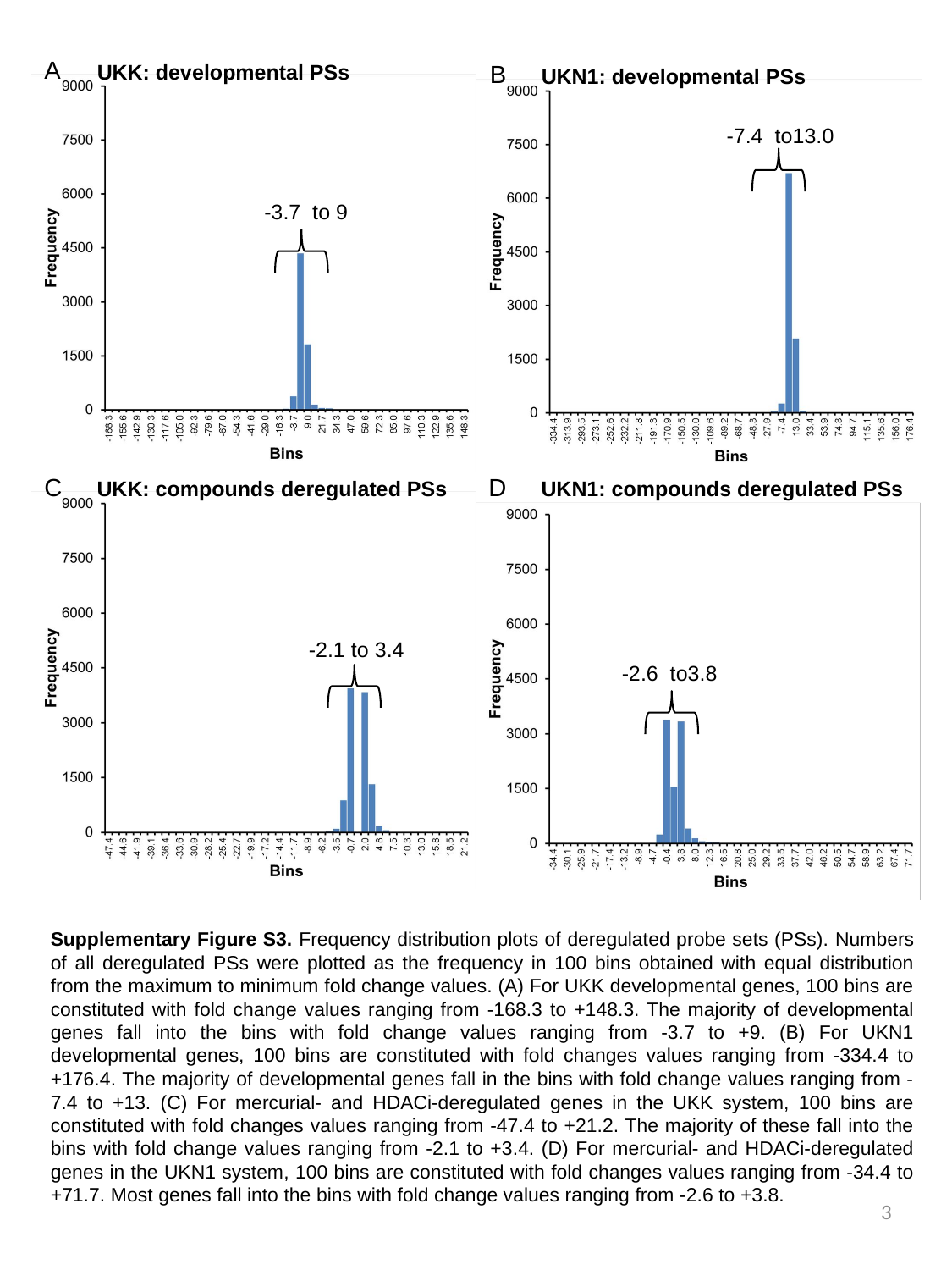

A
B
UKK: developmental PSs
UKN1: developmental PSs
-3.7 to 9
-7.4 to13.0
C
D
UKK: compounds deregulated PSs
UKN1: compounds deregulated PSs
-2.1 to 3.4
-2.6 to3.8
Supplementary Figure S3. Frequency distribution plots of deregulated probe sets (PSs). Numbers of all deregulated PSs were plotted as the frequency in 100 bins obtained with equal distribution from the maximum to minimum fold change values. (A) For UKK developmental genes, 100 bins are constituted with fold change values ranging from -168.3 to +148.3. The majority of developmental genes fall into the bins with fold change values ranging from -3.7 to +9. (B) For UKN1 developmental genes, 100 bins are constituted with fold changes values ranging from -334.4 to +176.4. The majority of developmental genes fall in the bins with fold change values ranging from -7.4 to +13. (C) For mercurial- and HDACi-deregulated genes in the UKK system, 100 bins are constituted with fold changes values ranging from -47.4 to +21.2. The majority of these fall into the bins with fold change values ranging from -2.1 to +3.4. (D) For mercurial- and HDACi-deregulated genes in the UKN1 system, 100 bins are constituted with fold changes values ranging from -34.4 to +71.7. Most genes fall into the bins with fold change values ranging from -2.6 to +3.8.
3

## Slide 4
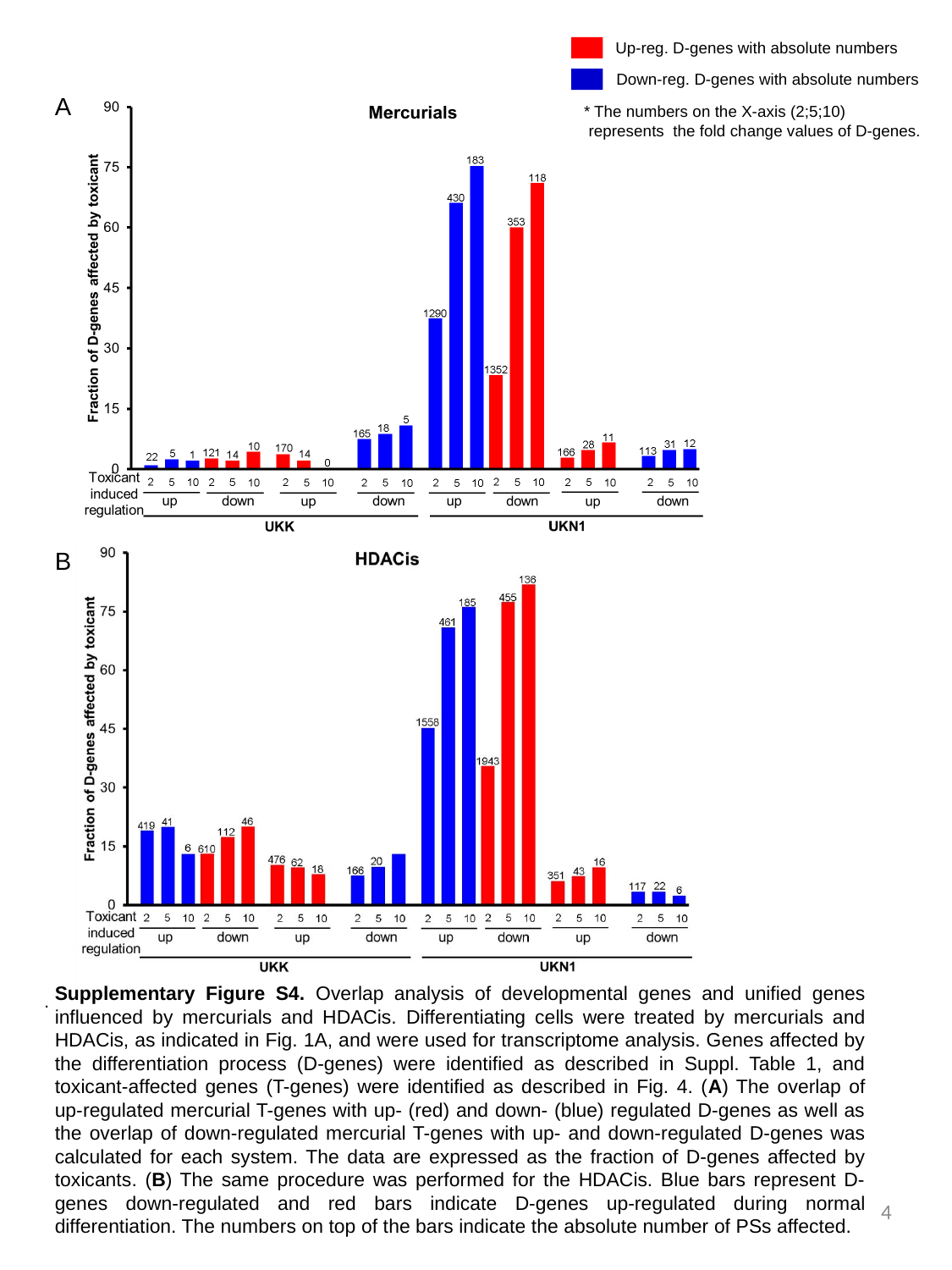

Up-reg. D-genes with absolute numbers
Down-reg. D-genes with absolute numbers
A
* The numbers on the X-axis (2;5;10)
 represents the fold change values of D-genes.
B
Supplementary Figure S4. Overlap analysis of developmental genes and unified genes influenced by mercurials and HDACis. Differentiating cells were treated by mercurials and HDACis, as indicated in Fig. 1A, and were used for transcriptome analysis. Genes affected by the differentiation process (D-genes) were identified as described in Suppl. Table 1, and toxicant-affected genes (T-genes) were identified as described in Fig. 4. (A) The overlap of up-regulated mercurial T-genes with up- (red) and down- (blue) regulated D-genes as well as the overlap of down-regulated mercurial T-genes with up- and down-regulated D-genes was calculated for each system. The data are expressed as the fraction of D-genes affected by toxicants. (B) The same procedure was performed for the HDACis. Blue bars represent D-genes down-regulated and red bars indicate D-genes up-regulated during normal differentiation. The numbers on top of the bars indicate the absolute number of PSs affected.
.
4

## Slide 5
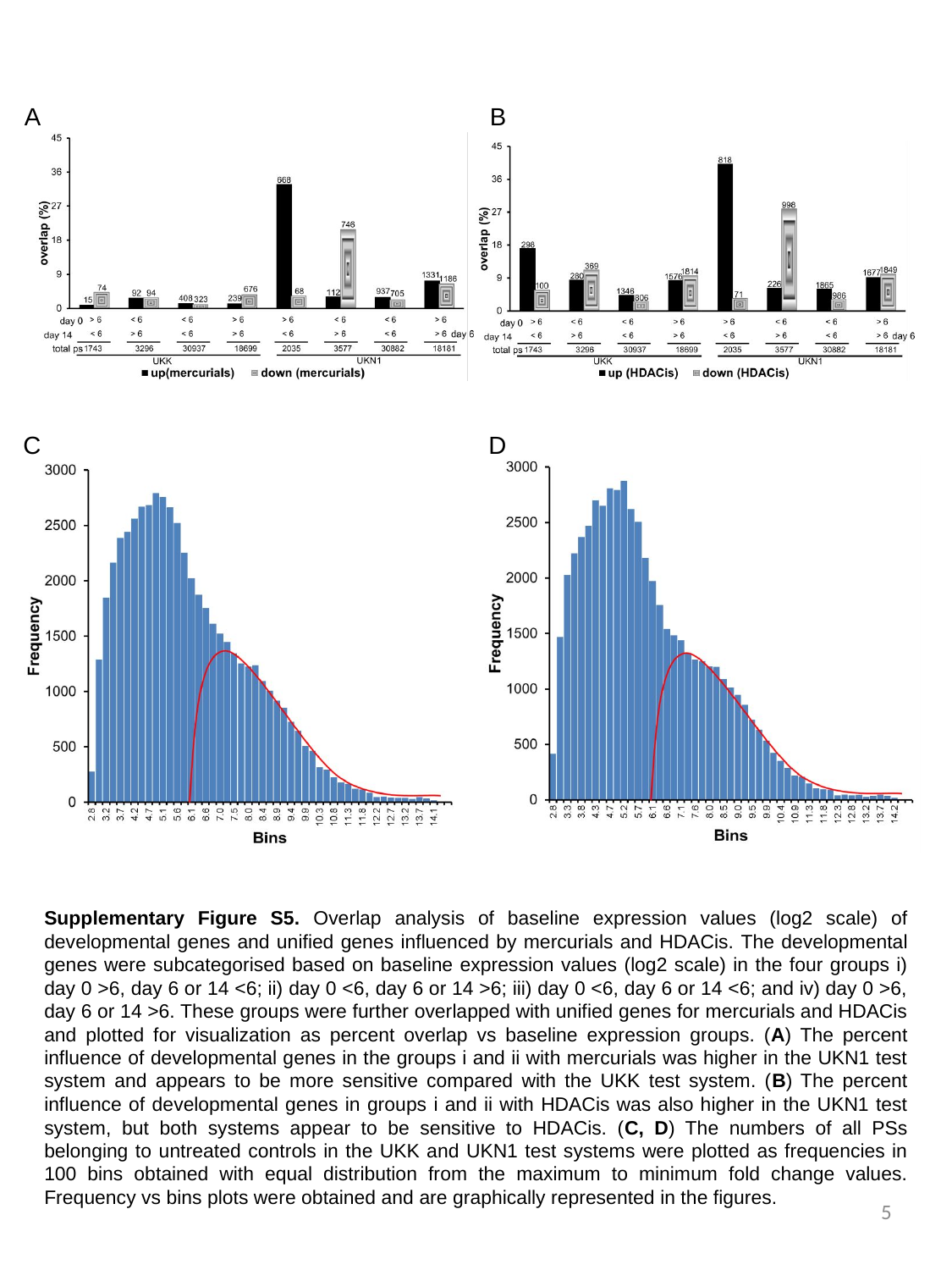

A
B
C
D
Supplementary Figure S5. Overlap analysis of baseline expression values (log2 scale) of developmental genes and unified genes influenced by mercurials and HDACis. The developmental genes were subcategorised based on baseline expression values (log2 scale) in the four groups i) day 0 >6, day 6 or 14 <6; ii) day 0 <6, day 6 or 14 >6; iii) day 0 <6, day 6 or 14 <6; and iv) day 0 >6, day 6 or 14 >6. These groups were further overlapped with unified genes for mercurials and HDACis and plotted for visualization as percent overlap vs baseline expression groups. (A) The percent influence of developmental genes in the groups i and ii with mercurials was higher in the UKN1 test system and appears to be more sensitive compared with the UKK test system. (B) The percent influence of developmental genes in groups i and ii with HDACis was also higher in the UKN1 test system, but both systems appear to be sensitive to HDACis. (C, D) The numbers of all PSs belonging to untreated controls in the UKK and UKN1 test systems were plotted as frequencies in 100 bins obtained with equal distribution from the maximum to minimum fold change values. Frequency vs bins plots were obtained and are graphically represented in the figures.
5

## Slide 6
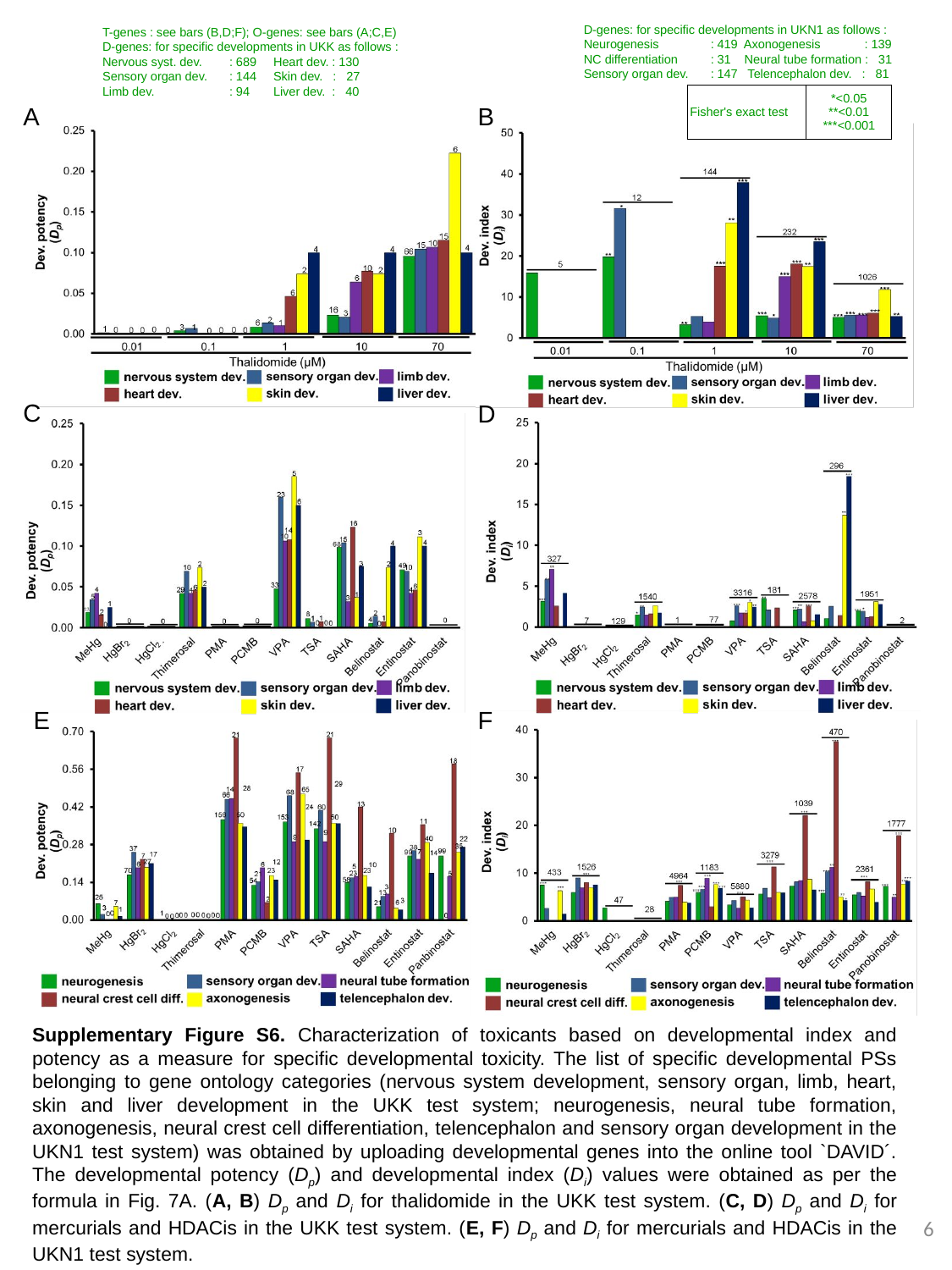

D-genes: for specific developments in UKN1 as follows :
Neurogenesis	: 419 Axonogenesis 	 : 139
NC differentiation 	: 31 Neural tube formation : 31
Sensory organ dev. 	: 147 Telencephalon dev. : 81
T-genes : see bars (B,D;F); O-genes: see bars (A;C,E)
D-genes: for specific developments in UKK as follows :
Nervous syst. dev.	: 689 Heart dev. : 130
Sensory organ dev. 	: 144 Skin dev. : 27
Limb dev. 	: 94 Liver dev. : 40
| Fisher's exact test | \*<0.05 \*\*<0.01 \*\*\*<0.001 |
| --- | --- |
A
B
C
D
E
F
Supplementary Figure S6. Characterization of toxicants based on developmental index and potency as a measure for specific developmental toxicity. The list of specific developmental PSs belonging to gene ontology categories (nervous system development, sensory organ, limb, heart, skin and liver development in the UKK test system; neurogenesis, neural tube formation, axonogenesis, neural crest cell differentiation, telencephalon and sensory organ development in the UKN1 test system) was obtained by uploading developmental genes into the online tool `DAVID´. The developmental potency (Dp) and developmental index (Di) values were obtained as per the formula in Fig. 7A. (A, B) Dp and Di for thalidomide in the UKK test system. (C, D) Dp and Di for mercurials and HDACis in the UKK test system. (E, F) Dp and Di for mercurials and HDACis in the UKN1 test system.
6

## Slide 7
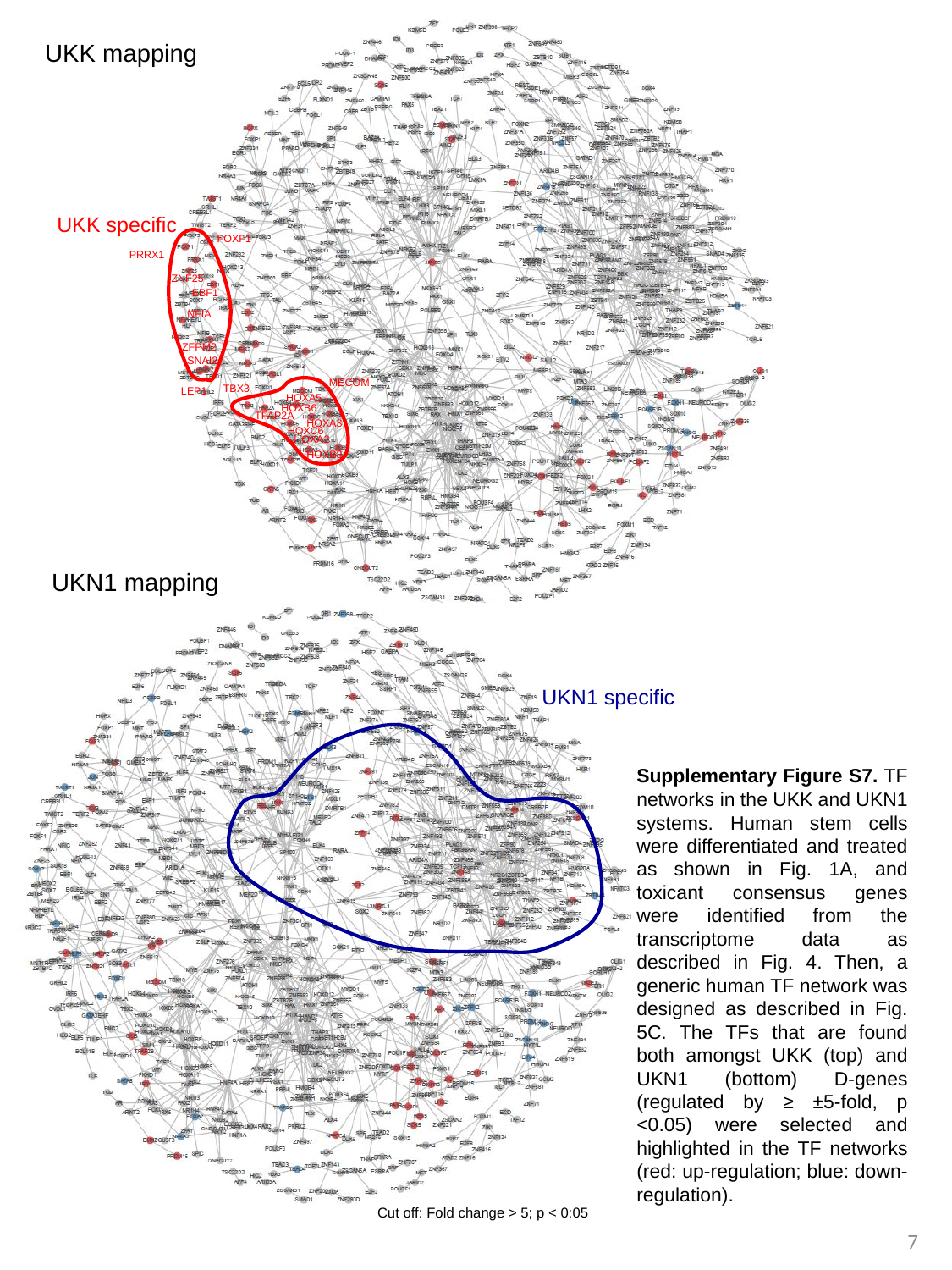

FOXF1
PRRX1
ZNF25
EBF1
NFIA
ZFPM2
SNAI2
MECOM
TBX3
LEP1
HOXA5
HOXB6
TFAP2A
HOXA3
HOXC6
HOXA2
HOXB8
UKK mapping
UKK specific
UKN1 mapping
UKN1 specific
Supplementary Figure S7. TF networks in the UKK and UKN1 systems. Human stem cells were differentiated and treated as shown in Fig. 1A, and toxicant consensus genes were identified from the transcriptome data as described in Fig. 4. Then, a generic human TF network was designed as described in Fig. 5C. The TFs that are found both amongst UKK (top) and UKN1 (bottom) D-genes (regulated by ≥ ±5-fold, p <0.05) were selected and highlighted in the TF networks (red: up-regulation; blue: down-regulation).
Cut off: Fold change > 5; p < 0:05
7
